# Supplementary material for: Essential Oils Biofilm Modulation Activity and Machine Learning Analysis on Pseudomonas aeruginosa Isolates from Cystic Fibrosis Patients
Source: Microorganisms. 2022 Apr 24;10(5):887. doi: 10.3390/microorganisms10050887 (PMC9145053; doi:10.3390/microorganisms10050887)

microorganisms

MDPI

SUPPLEMENTARY MATERIAL

Essential oils biofilm modulation activity and machine learning analysis on *Pseudomonas aeruginosa* isolates from cystic fibrosis patients.

Marco Artini [1,†], Rosanna Papa[1,†], Filippo Sapienza [2,3], Mijat Božović [4], Gianluca Vrenna[1], Vanessa Tuccio Guarna Assanti[5], Manuela Sabatino [2,3], Stefania Garzoli [3], Ersilia Vita Fiscarelli [5], Rino Ragno [2,3,6,*] and Laura Selan [1,*]

[1] Department of Public Health and Infectious Diseases, Sapienza University, p.le Aldo Moro 5, 00185 Rome, Italy. marco.artini@uniroma1.it, rosanna.papa@uniroma1.it, gianluca.vrenna@uniroma1.it, laura.selan@uniroma1.it
[2] Rome Center for Molecular Design, Department of Drug Chemistry and Technology, Sapienza University, p.le Aldo Moro 5, 00185 Rome, Italy. rino.ragno@uniroma1.it, filippo.sapienza@uniroma1.it, manuela.sabatino@uniroma1.it, stefania.garzoli@uniroma1.it
[3] Department of Drug Chemistry and Technology, Sapienza University, p.le Aldo Moro 5, 00185 Rome, Italy; rino.ragno@uniroma1.it, filippo.sapienza@uniroma1.it, manuela.sabatino@uniroma1.it
[4] Faculty of Natural Sciences and Mathematics, University of Montenegro, Džordža Vašingtona bb, 81000 Podgorica, Montenegro; mijatboz@ucg.ac.me
[5] Paediatric and Laboratory Department. Children’s Hospital and Institure Research Bambino Gesù, Italy; evita.fiscarelli@opbg.net
[6] Società Italiana Ricerca Oli Essenziali, Viale Regina Elena, 299 00161 Roma. rino.ragno@uniroma1.it

Table S1. Qualitative descriptors used for the unsupervised Machine Learning clusterization of *P. aeruginosa* strains.

**Descriptor Name**	**Parameter**
Type of sample	Esp: sputum; AT: hypopharyngeal suction
Type of strain	*P. aeruginosa*; *P. aeruginosa* multi-drug resistant
Phenotype	wrinkled colony surface; mucoid colony; irregular colony edges; smooth phenotype
Carbapenems Sensitivity	Resistant, Susceptible or Intermediate
Meropenem Sensitivity	Resistant, Susceptible or Intermediate
Imipenem Sensitivity	Resistant, Susceptible or Intermediate
Piperacillin/tazobactam Sensitivity	Resistant, Susceptible or Intermediate
Aminoglycosides Sensitivity	Resistant, Susceptible or Intermediate
Quinolones Sensitivity	Resistant, Susceptible or Intermediate
Monobactam	Resistant, Susceptible or Intermediate
Cephalosporins	Resistant, Susceptible or Intermediate
Colistin	Resistant, Susceptible or Intermediate
First Infection	Positive or negative
Early Infection	Positive or negative
Late Infection	Positive or negative
S. aureus co-infection	Positive or negative
cftr gene mutation	N1303K/3849+10kbC>T<br>F508del/F508del<br>W1282X/2789+5G->A<br>F508del/E193K
Biofilm formation	Strong, moderate or weak

Table S2. Phenotypical and genotypical characterization of 6 representative strains of *P. aeruginosa*.

**ID pt**	**21**	**23**	**24**	**24**	**28**	**29**
**ID**	22P	25P	26P	27P	37P	39P
**SAM**	ESP	AT	TF	AT	AT	ESP
**date**	1/11/2017	9/3/2010	8/27/2008	1/31/2017	2/22/2017	1/25/2017
**Str**	PA MDR	PA	PA	PA	PA	PA MDR
**Ph**	w	sm	i	sm	m	m
**CAR**	R	S	S	S	MP S/ IP R	R
**PTC**	S	S	S	S	S	R
**AM**	R	I	S	S	R	R
**QUIN**	R	S	S	S	S	R
**MB**	S	I	I	S	S	I
**CEF**	R	S	S	S	R	R
**COL**	S	S	S	S	S	S
**1St**		X	X			
**E**						
**L**	X			X	X	X
**CSA**				X		
**GEN**	N1303K/ 3849+10kbC>T	F508del/ F508del	W1282X/ 2789+5G->A	W1282X/ 2789+5G->A	F508del/ E193K	F508del/ F508del

The 6 *P. aeruginosa* clinical isolates and their characterization by several properties. **ID pt**: patient identification; **ID**: strain code; **SAM**: Sample; **Date**: Date of collection; **Str**: Strain; **P**h: Phenotype; **CAR**: Carbapenems; **MP**: Meropenem; **IP**: Imipenem; **PTC**: Piperacillin/tazobactam; **AM**: Aminoglycosides; **QUIN**: Quinolones; **MB**: Monobactam; **CEF**: Cephalosporins; **COL**: Colistin; **1 St**: *P. aeruginosa* first isolate; **E**: *P. aeruginosa* early isolate; **L**: *P. aeruginosa* late isolate; **CSA**: *S. aureus* co-infection; **Esp**: sputum; **AT**: hypopharyngeal suction; **PA**: *P. aeruginosa*; **PA MDR**: *P. aeruginosa* multi-drug resistant; **w**: wrinkled colony surface; **m**: mucoid colony; **i**: irregular colony edges; **sm**: smooth phenotype; **R**: Resistant; **S**: Susceptible; **I**: Intermediate; **X**: denotes positive for the feature; **GEN**: Genotype

Table S3. Essential oil IDs and associated EO names.

EO ID	EO Name	EO ID	EO Name
EO1	Chamomile Morocco	EO32	Birch
EO2	Sage Sclarea	EO33	Fennel
EO3	Salvia Officinalis	EO34	Cedar Fruit
EO4	Red Thyme	EO35	Lemon
EO5	Tea Tree Oil	EO36	Roman Chamomile
EO6	Melissa Oiio Essential	EO37	Savory
EO7	Pinus Mugo	EO38	Rosemary
EO8	Geranium Bourbon	EO39	Ceylon Cinnamon Peel
EO9	Oregano	EO40	Eucaliptus Globulus
EO10	Ylang Ylang	EO41	Sweet Orange
EO11	Coriander	EO42	Niaouly
EO12	Lavandula Angustifoglia	EO43	Artemisia
EO13	Myrtle	EO44	Cajeput
EO14	Garlic	EO45	Black Pepper
EO15	Cardamom	EO46	White Thyme
EO16	Mandarin	EO47	Marjoram
EO17	Hyssop	EO48	Cloves
EO18	Grapefruit	EO49	Cypress
EO19	Cymbopogon	EO50	Nutmeg Natural
EO20	Pinus Sibirica	EO51	Peppermint
EO21	Camphor	EO52	Verbena officinalis
EO22	Cadè	EO53	Basil
EO23	Cedar Leaves	EO54	Cymbopogon martinii
EO24	Ginger	EO55	Laurel
EO25	Cumin	EO56	Anise
EO26	Patchouli	EO57	Incense
EO27	Bitter Orange	EO58	Mentha Suaveolens
EO28	Eucalyptus	EO59	Coridotthymus Capitatus
EO29	Pinus Silvester	EO60	Thymus Vulgaris
EO30	Bergamot	EO61	Origanum Hirtum
EO31	Juniper		

Table S4. List of sistematically DA settings varied during ML hyperparameters optimization

Parameter	Settings	Description
preprocess_fns	aug10, aug20, baug10, baug20	This is the function tha apply the data augmentation The numbers 10 or 20 indicates the times each component percentage is increased or decreased up to 15%. Baug and aug indicated whether the dataset will be balanced or not.
component_min	0, 2, 4, 6	This represent the number of least occurrences for EO's components to be maitained in the training set
pca	60 %, 80 %, 90 %, 100 %	Amount of variance extracted with the PCA. 100% indicate tha the whole EOs' data matrix was used but trasformed to avoid the usage of correlate variables.

Table S5. List of hyperparameters setting used for the preliminary ML models' through random search optimization

Algorithm	Parameters	Settings	#	Total Combinations
	class_weight	list_weight	17	
RF	n_estimators	from 1 to 200, step 10	20	136000
	max_depth	from 1 to 200, step 10	20	
	min_samples_leaf	from 1 to 200, step 10	20	
	n_estimators	from 1 to 200, step 10	20	
GB	max_depth	from 1 to 200, step 10	20	8000
	min_samples_leaf	from 1 to 200, step 10	20	
	class_weight	list_weight	17	
	C	from 0.001 to 100, incrementing 20%	100	
LR	penalty	l1, l2, elasticnet	3	25500
	solver	newton-cg, lbfgs, liblinear, sag, saga	5	
	max_iter	10000	1	
	class_weight	list_weight	17	
SV	C	from 0.001 to 100, incrementing 20%	100	
	kernel	linear, poly, rbf, sigmoid	4	6800
	probability	TRUE	1	
	class_weight	list_weight	17	
	criterion	gini, entropy	2	
	splitte<r	best, random	2	
DT	max_depth	from 1 to 20, step 5	4	
	min_samples_split	from 1 to 20, step 5	4	17408
	min_samples_leaf	from 1 to 20, step 5	4	
	max_features	auto, sqrt, log2, None	4	
	n_neighbors	from 1 to 30, step 5	6	
	weights	uniform, distance	2	
	algorithm	auto, ball_tree, kd_tree, brute	4	
KNN	leaf_size	from 1 to 30, step 5	6	2304
	metric	minkowski, euclidean, manhattan, chebyshev	4	
	metric_params	None	1	
	p	1, 2	2	

Table S6. List of weight for the class_weight hyperparamenters in Table SM 6. Data are presented as python dictionaries

Combination	Balancing for class A	Balancing for class B	Balancing %
**1**	{0:1.0, 1:1.0},		**0**
**2**	{0:1.0, 1:1.1},	{0:1.1, 1:1.0},	**10**
**3**	{0:1.0, 1:1.2},	{0:1.2, 1:1.0},	**20**
**4**	{0:1.0, 1:1.3},	{0:1.3, 1:1.0},	**30**
**5**	{0:1.0, 1:1.4},	{0:1.4, 1:1.0},	**40**
**6**	{0:1.0, 1:1.5},	{0:1.5, 1:1.0},	**50**
**7**	{0:1.0, 1:2.0},	{0:2.0, 1:1.0},	**100**
**8**	{0:1.0, 1:2.5},	{0:2.5, 1:1.0},	**150**
**9**	{0:1.0, 1:3.0},	{0:3.0, 1:1.0}]	**200**

Table S7. List of hyperparameters setting used for the models’ refining through random search optimization

Algorithm	Parameters	Settings	#	Total Combinations
	class_weight	list_weight	17	
RF	n_estimators	from 1 to 200, step 1	200	136000000
	max_depth	from 1 to 200, step 1	200	
	min_samples_leaf	from 1 to 200, step 1	200	
	n_estimators	from 1 to 200, step 1	200	
GB	max_depth	from 1 to 200, step 1	200	8000000
	min_samples_leaf	from 1 to 200, step 1	200	
	class_weight	list_weight	17	
	C	from 0.001 to 100, incrementing 20%	100	
LR	penalty	l1, l2, elasticnet	3	25500
	solver	newton-cg, lbfgs, liblinear, sag, saga	5	
	max_iter	10000	1	
	class_weight	list_weight	17	
SV	C	from 0.001 to 100, incrementing 20%	100	
	kernel	linear, poly, rbf, sigmoid	4	6800
	probability	True	1	
	class_weight	list_weight	17	
	criterion	gini, entropy	2	
	splitte<r	best, random	2	
DT	max_depth	from 1 to 20, step 1	20	2176000
	min_samples_split	from 1 to 20, step 1	20	
	min_samples_leaf	from 1 to 20, step 1	20	
	max_features	auto, sqrt, log2, None	4	
	n_neighbors	from 1 to 30, step 1	30	
	weights	uniform, distance	2	
	algorithm	auto, ball_tree, kd_tree, brute	4	
KNN	leaf_size	from 1 to 30, step 1	30	57600
	metric	minkowski, euclidean, manhattan, chebyshev	4	
	metric_params	None	1	
	p	1, 2	2	

Table S8. Antimicrobial activity of EOs listed in Table SM1, on representative clinical and reference strains of *P. aeruginosa*.

EOs ID	PaO1	PA14	22P	25P	26P	27P	37P	39P
**EO1**	I	I	I	I	I	I	I	I
**EO2**	I	I	I	I	I	I	I	I
**EO3**	I	I	A	I	I	I	A	I
**EO4**	I	I	A	I	I	I	A	I
**EO5**	I	I	I	I	I	I	I	I
**EO6**	I	I	I	I	I	I	I	I
**EO7**	I	I	I	I	I	I	I	I
**EO8**	I	I	I	I	I	I	I	I
**EO9**	I	A	A	A	A	A	A	A
**EO10**	I	I	I	I	I	I	I	I
**EO11**	I	I	I	I	I	I	I	I
**EO12**	I	I	I	I	I	I	I	I
**EO13**	I	I	I	I	I	I	I	I
**EO14**	A	A	I	A	I	A	A	A
**EO15**	I	I	I	I	I	I	I	I
**EO16**	I	I	I	I	I	I	I	I
**EO17**	I	I	I	I	I	I	I	I
**EO18**	I	I	I	I	I	I	I	I
**EO19**	I	I	I	I	I	I	I	I
**EO20**	I	I	I	I	I	I	I	I
**EO21**	I	I	I	I	I	I	I	I
**EO22**	A	A	A	A	A	A	A	A
**EO23**	I	I	I	I	I	I	I	I
**EO24**	I	I	I	I	I	I	I	I
**EO25**	I	I	I	I	I	I	I	I
**EO26**	I	I	I	I	I	I	I	I
**EO27**	I	I	I	I	I	I	I	I
**EO28**	I	I	I	I	I	I	I	I
**EO29**	I	I	I	I	I	I	I	I
**EO30**	I	I	I	I	I	I	I	I
**EO31**	I	I	I	I	I	I	I	I
**EO32**	A	A	A	A	A	A	A	A
**EO33**	I	I	I	I	I	I	I	I
**EO34**	I	I	I	I	I	I	I	I
**EO35**	I	I	I	I	I	I	I	I
**EO36**	I	I	I	I	I	I	I	I
**EO37**	I	I	A	I	I	I	A	A
**EO38**	I	I	I	I	I	I	A	I
**EO39**	A	A	A	A	A	A	A	A
**EO40**	I	I	I	I	I	I	I	I

**EO41**	I	I	I	I	I	I	I	I
**EO42**	I	I	I	I	I	I	I	I
**EO43**	I	I	I	I	I	I	I	I
**EO44**	I	I	I	I	I	I	I	I
**EO45**	I	I	I	I	I	I	I	I
**EO46**	I	I	I	I	I	I	A	A
**EO47**	I	I	I	I	I	I	I	I
**EO48**	I	A	I	I	I	I	I	A
**EO49**	I	I	I	I	I	I	I	I
**EO50**	I	I	I	I	I	I	I	I
**EO51**	I	I	I	I	I	I	I	I
**EO52**	I	I	I	I	I	I	I	I
**EO53**	I	I	I	I	I	I	I	I
**EO54**	I	I	I	I	I	I	I	I
**EO55**	I	I	I	I	I	I	I	I
**EO56**	I	I	I	I	I	I	I	I
**EO57**	I	I	I	I	I	I	I	I
**EO58**	I	I	I	I	I	I	I	I
**EO59**	I	I	I	I	I	I	A	A
**EO60**	I	I	I	I	I	I	A	A
**EO61**	I	I	I	I	I	I	I	A

I: Inactive; A: Antibacterial

Table S9. Compositions of the 61 essential oils used in the study

	EO1
.τ.-muurolol	2.1
artemisia alcohol	1.3
borneol	1.2
borneol, butyrate	4.3
β-caryophyllene	1.0
caryophyllene oxide	1.3
cis-3-pinanone	0.3
cis-β-farnesene	5.2
eucalyptol	2.0
germacrene d	4.5
isobornyl 3-methylbutanoate	3.1
limonene	6.7
linalool	0.3
myrtenol	0.4
o-cymene	0.2
pinocarveol	7.4
sativene	0.9
spathulenol	4.0
terpinen-4-ol	0.4
trans-2,7-dimethyl-4,6-octadien-2-ol	33.2
yomogi alcohol	1.3
α-pinene	14.4
β-myrcene	1.4
δ-cadinene	0.5
δ-elemene	1.7
τ-cadinol	0.5

	EO2
β-caryophyllene	2.0
caryophyllene oxide	2.3
cis-geraniol	1.7
geranyl acetate	3.5
linalool	19.9
linalyl anthranilate	60.0
nerol acetate	2.2
trans-linalool oxide	2.1
α-bergamotene	0.8
α-copaene	0.6
α-terpineol	3.8
β-bisabolene	0.7
β-myrcene	0.3

	EO3
alcanfor	21.3
borneol	4.6
bornyl acetate	2.6
camphene	3.5
β-caryophyllene	6.8
caryophyllene oxide	0.3
chrysanthone	25.1
eucalyptol	11.7
humulene	7.5
humulene epoxide 2	0.3
limonene	1.3
o-cymene	1.3
terpinen-4-ol	0.6
thujone	7.8
viridiflorol	0.2
α-pinene	3.0
β-pinene	1.4
γ-terpinene	0.5

	EO4
borneol	1.5
carvacrol	7.2
β-caryophyllene	2.3
caryophyllene oxide	0.6
eucalyptol	0.3
limonene	0.3
linalool	5.2
methyl thymyl ether	0.2
p-cymene	10.5
terpinen-4-ol	1.8
thymol	66.3
α-citral	0.1
α-pinene	0.4
α-terpinene	0.4
α-terpineol	0.2
β-myrcene	0.5
γ-terpinene	2.3

	EO5
eucalyptol	14.9
globulol	0.4
limonene	2.0
linalool, oxide	0.2
longifolene	0.2
o-cymene	3.5
terpinen-4-ol	37.5
terpinolene	1.7
viridiflorene	1.1
α-gurjunene	0.2
α-pinene	11.1
α-terpinene	4.6
α-terpineol	8.1
β-myrcene	0.2
β-pinene	2.5
γ-terpinene	11.8

	EO6
6-methyl-5-hepten-2-one	0.3
β-caryophyllene	31.4
caryophyllene oxide	5.5
cis-geraniol	2.5
citronellal	26.3
eucalyptol	0.7
humulene epoxide 2	0.9
limonene	1.6
linalool	0.8
α-citral	28.2
α-copaene	0.7
γ-cadinene	0.6
δ-cadinene	0.5

	EO7
3-carene	10.8
bornyl acetate	13.4
calamenene	0.7
carvone	0.3
β-caryophyllene	21.4
cis-verbenol	0.5
crypton	3.7
cumaldehyde	0.5
humulene	1.2
isopinocarveol	0.5
limonene	10.9
linalool	0.2
o-cymene	2.1
p-cymen-8-ol	0.8
trans-2-caren-4-ol	0.6
α-copaene	0.9
α-muurolene	1.1
α-pinene	12.5
α-terpineol	0.4
β-cubebene	1.2
β-phellandrene	7.0
β-pinene	7.6
δ-cadinene	1.6

	EO8
1-indanone	3.8
2,6-dimethyl-2,6-octadiene	0.6
2-pentadecanone, 6,10,14-trimethyl	0.4
acetic acid	5.0
cis-geraniol	12.9
citronellol	26.3
citronellyl butyrate	1.5
citronellyl formate	8.0
geraniol formate	3.8
geranyl isobutyrate	1.2
geranyl propionate	0.8
heptanoic acid	1.2
hexanoic acid	0.6
isomenthone	4.6
limonene	8.4
linalool	9.6
linalyl anthranilate	6.8
o-cymene	1.3
rose oxide	0.9
α-pinene	0.5
β-myrcene	0.4
β-pinene	1.2

	EO9
borneol	0.4
carvacrol	76.5
β-caryophyllene	6.7
caryophyllene oxide	2.3
eucalyptol	0.4
limonene	0.5
linalool	2.4
p-cymene	6.8
terpinen-4-ol	0.5
α-pinene	0.4
α-terpinene	0.3
β-myrcene	0.8
γ-terpinene	1.9

	EO10
acetic acid, cinnamyl ester	1.0
calamenene	0.3
β-caryophyllene	15.5
cis-geraniol	1.5
cis-β-farnesene	13.9
eucalyptol	0.2
eugenol	0.6
farnesol	2.6
farnesyl acetate	3.5
geranyl acetate	12.2
germacrene d	18.2
humulene	4.3
linalool	10.4
methyl benzoate	2.1
p-methylanisole	3.3
α-cadinol	1.1
α-copaene	1.2
β-elemene	0.5
β-ylangene	0.5
γ-cadinene	1.0
γ-muurolene	1.8
δ-cadinene	3.8
τ-cadinol	0.6

	EO11
3,7-octadiene-2,6-diol, 2,6-dimethyl-	0.2
benzyl benzoate	6.4
camphene	0.4
camphor	5.7
cis-geraniol	1.7
eucalyptol	0.4
geranyl acetate	5.2
limonene	1.7
linalool	66.7
o-cymene	2.5
salicyclic acid, benzyl ester	2.2
terpinen-4-ol	0.2
trans-linalool oxide	0.6
α-pinene	3.4
α-terpineol	0.8
β-myrcene	0.5
β-pinene	0.3
γ-terpinene	0.9

	EO12
1-octen-3-ol	0.3
3-octanol	0.3
3-octanone	0.9
borneol	1.4
butanoic acid, hexyl ester	0.5
β-caryophyllene	2.7
cis-geraniol	0.4
cis-β-farnesene	1.9
cis-β-ocimene	1.0
eucalyptol	2.4
lavandulyl acetate	4.3
limonene	0.5
linalool	34.7
linalyl acetate	41.4
terpinen-4-ol	3.8
α-pinene	0.2
α-terpineol	1.3
β-myrcene	0.7
β-ocimene	1.3

	EO13
1,4-dihydroxy-p-menth-2-ene	0.3
3-carene	0.2
β-caryophyllene	2.2
cis-geraniol	0.7
eucalyptol	40.8
geranyl acetate	9.7
humulene	0.5
isobutyl 2-methylbutyrate	0.5
isobutyl isobutyrate	0.3
limonene	7.1
linalool	3.7
linalyl anthranilate	1.2
methyleugenol	2.0
o-cymene	5.3
p-cymen-8-ol	0.7
pinocarveol	0.2
terpinen-4-ol	0.6
α-pinene	17.4
α-terpineol	3.9
α-terpineol acetate	1.9
β-myrcene	0.2
β-pinene	0.2
γ-elemene	0.2

	EO14
1,2-dithiolane	0.5
1,2-dithiolane, 1,1-dioxide	2.8
3-vinyl-1,2-dithiacyclohex-4-ene	2.6
diallyl disulphide	57.6
diallyl sulfide	3.8
dimethyl trisulfide	0.5
disulfide, methyl 2-propenyl	2.7
eucalyptol	0.3
limonene	0.5
trans-3,5-diethyl-1,2,4-trithiolane	0.3
trisulfide, di-2-propenyl	2.1
trisulfide, methyl 2-propenyl	26.1

	EO15
cis-geraniol	1.1
cis-β-terpineol	0.7
eucalyptol	34.7
limonene	2.0
linalool	5.0
linalyl anthranilate	8.2
nerolidol	1.3
o-cymene	0.4
sabinene	0.3
α-pinene	1.3
α-terpineol	1.9
α-terpineol acetate	41.8
β-myrcene	1.1
β-pinene	0.3

	EO16
limonene	66.7
o-cymene	7.6
sabinene	0.2
terpinolene	0.9
α-pinene	2.4
α-terpineol	0.3
β-myrcene	1.6
β-pinene	1.3
γ-terpinene	19.0

	EO17
4-methoxycinnamaldehyde	1.1
borneol	1.1
β-caryophyllene	1.3
caryophyllene oxide	0.6
cis-3-pinanone	34.1
dhs activator	0.6
elemol	1.1
estragole	0.2
eucalyptol	0.6
limonene	1.4
linalool	18.0
myrtenal	0.5
myrtenol	1.0
o-cymene	0.5
pinocarveol	0.3
sabinene	0.6
spathulenol	1.1
terpinen-4-ol	0.9
trans-pinocamphone	18.9
α-pinene	8.6
α-terpineol	0.8
β-myrcene	0.3
β-pinene	6.4

	EO18
p-mentha-2,8-dien-1-ol	1.9
carveol	1.9
carvone	4.7
caryophyllene oxide	0.4
cis-carveol	3.3
cis-limonene oxide	3.5
limonene	78.2
limonene-1,2-diol	0.7
o-cymene	0.3
trans-limonene oxide	2.4
trans-p-mentha-2,8-dienol	2.1
α-pinene	0.4
β-myrcene	0.3

	EO19
.τ.-muurolol	0.2
β-caryophyllene	0.2
citronellal	38.8
citronellol	19.0
citronellyl acetate	2.9
elemol	2.5
eugenol	0.9
geraniol	24.8
germacrene D	0.7
germacrene-D-4-ol	0.3
isopulegol	0.7
linalool	0.4
α-cadinol	0.4
α-citral	2.0
β-citral	1.2
β-elemene	0.9
β-myrcene	3.0
γ-muurolene	0.3
δ-cadinene	0.9

	EO20
borneol	2.7
bornyl acetate	55.0
camphene	18.1
camphor	0.4
β-caryophyllene	0.9
caryophyllene oxide	1.3
crypton	0.6
humulene	0.5
humulene epoxide 2	0.6
limonene	3.9
o-cymene	0.4
p-cymen-7-ol	0.2
p-cymen-8-ol	0.7
santene	1.0
tricyclene	1.5
α-bisabolol	0.4
α-pinene	8.4
α-terpineol	0.4
β-bisabolene	0.2
β-phellandrene	1.1
β-pinene	1.6

	EO21
1,4-dihydroxy-p-menth-2-ene	0.5
camphene	0.4
estragole	0.3
eucalyptol	57.0
limonene	22.8
m-cymene	13.4
palmitic acid	1.6
sabinene	0.6
α-pinene	0.4
β-pinene	3.1

	EO22
2,2,7,7-tetramethyltricyclo[6.2.1.0~1,6~]undeca-3,5,9-triene	2.4
2-methoxy-4-propylphenol	3.0
2-methoxy-phenol	4.2
2-methyl-phenol	1.3
3-methyl-1,2-cyclopentanedione	1.2
4-ethyl-2-methoxyphenol	7.3
8,14-cedranoxide	0.3
calamenene	17.4
β-caryophyllene	1.0
cedrene	8.8
cedrol	0.5
creosol	9.3
cubenol	2.7
humulene	1.6
isoeugenol	1.9
isoledene	3.7
m-cresol	1.4
phenol	1.0
α-cadinol	0.5
α-calacorene	6.3
α-curcumene	1.1
α-muurolene	4.6
δ-cadinene	18.6

	EO23
bornyl acetate	0.6
camphene	0.3
camphor	3.8
chrysanthone	88.5
eucalyptol	0.3
fenchone	2.7
limonene	0.3
sabinene	0.3
terpinen-4-ol	0.7
thujone	1.8
α-pinene	0.4
β-myrcene	0.3

	EO24
6-methyl-5-hepten-2-one	0.6
borneol	1.1
camphene	5.7
cis-geraniol	0.4
elemol	0.5
eucalyptol	7.0
limonene	1.1
linalool	0.3
nerolidol	0.6
zingiberene	29.9
α-bergamotene	0.4
α-copaene	0.6
α-curcumene	31.6
α-farnesene	4.3
α-pinene	1.7
α-terpineol	0.7
β-bisabolene	10.6
β-elemene	1.2
β-eudesmol	0.3
β-myrcene	0.6
γ-muurolene	0.7

	EO25
2-caren-10-ol	28.1
carotol	1.3
carvacrol	0.3
β-caryophyllene	0.7
caryophyllene oxide	0.5
cis-β-farnesene	0.9
cumaldehyde	37.9
limonene	0.3
p-cymen-7-ol	0.5
p-cymen-8-ol	0.2
p-cymene	7.6
sabinene	0.2
α-phellandrene	1.0
α-pinene	0.3
β-myrcene	0.4
β-phellandrene	0.3
β-pinene	5.6
γ-terpinene	13.9

	EO26
β-caryophyllene	3.2
caryophyllene oxide	0.6
globulol	1.1
ledene oxide-(I)	0.4
patchouli alcohol	45.4
seychellene	6.8
α-guaiene	15.3
α-panasinsene	0.3
α-patchoulene	5.9
β-guaiene	19.0
β-patchoulene	2.1

	EO27
decanal	0.3
limonene	95.1
linalool	0.4
linalyl acetate	0.6
sabinene	0.2
α-pinene	0.5
β-pinene	2.7
γ-terpinene	0.1

	EO28
carvone	0.3
eucalyptol	83.9
limonene	6.1
p-cymene	8.1
α-pinene	1.1
β-myrcene	0.3
β-pinene	0.2

	EO29
3-carene	16.6
bornyl acetate	1.4
camphene	0.9
β-caryophyllene	16.7
caryophyllene oxide	3.5
cis-verbenol	0.2
humulene	1.2
humulene epoxide 2	0.3
limonene	11.2
longifolene	2.0
myrtenol	0.2
o-cymene	0.5
p-cymen-8-ol	1.6
pinocarveol	0.4
terpinolene	2.0
α-cubebene	0.5
α-pinene	22.8
α-terpineol	0.9
β-pinene	17.1

	EO30
carvone	0.4
cis-carveol	0.3
limonene	26.1
limonene oxide, cis-	0.3
linalool	13.8
linalyl anthranilate	45.3
nerol acetate	0.7
o-cymene	6.5
sabinene	0.5
α-pinene	0.7
β-bisabolene	0.2
β-myrcene	0.7
β-pinene	4.6

	EO31
(E)-β-farnesene	0.6
4-epi-cubebol	1.1
β-caryophyllene	13.7
caryophyllene oxide	2.9
humulene	3.6
limonene	7.5
o-cymene	3.0
p-cymen-8-ol	0.6
sabinene	5.9
spathulenol	0.8
terpinen-4-ol	6.3
terpinolene	0.9
trans-calamenene	0.9
α-cadinol	0.4
α-copaene	1.0
α-cubebene	0.9
α-muurolene	0.8
α-pinene	27.1
α-terpineol	0.6
β-elemene	3.6
β-myrcene	9.3
β-pinene	3.6
γ-elemene	2.0
γ-muurolene	1.5
γ-terpinene	1.0
δ-cadinene	0.4

	EO32
1,8-dimethylnaphthalene	4.6
2,2,7,7-tetramethyltricyclo[6.2.1.0~1,6~]undeca-3,5,9-triene	1.7
2-methoxy-4-propylphenol	2.7
2-methoxy-phenol	6.6
2-methyl-phenol	1.2
3-methyl-1,2-cyclopentanedione	0.8
4-epi-cubebol	2.8
benzocycloheptatriene	0.9
cadalene	5.5
cadina-1(2),4-diene	2.7
calamenene	18.1
cedrene	8.2
creosol	7.2
dihydrocurcumene	3.4
isoeugenol	1.6
m-cresol	1.3
α-cadinol	0.6
α-calacorene	5.8
α-muurolene	4.4
β-cadinene	0.7
δ-cadinene	18.5
δ-cadinol	0.8

	EO33
anethole	78.1
camphor	0.2
estragole	2.2
fenchone	3.4
limonene	3.4
o-cymene	0.4
p-acetonylanisole	4.7
p-anisaldehyde	6.3
α-bergamotene	0.1
α-pinene	1.0
β-myrcene	0.2

	EO34
carveol	0.4
carvone	1.4
β-caryophyllene	0.2
caryophyllene oxide	0.4
cis-p-mentha-2,8-dien-1-ol	0.4
geranic acid	1.0
limonene	32.2
limonene oxide, cis-	0.7
limonene oxide, trans-	0.5
linalool	6.4
linalyl anthranilate	25.0
nerol acetate	1.6
o-cymene	17.4
sabinene	0.2
trans-p-mentha-2,8-dienol	0.4
α-bergamotene	0.3
α-citral	4.8
α-pinene	0.4
β-bisabolene	0.5
β-citral	2.1
β-myrcene	0.4
β-pinene	3.2

	EO35
carveol	0.3
caryophyllene oxide	0.4
cis-geraniol	2.2
cis-p-mentha-2,8-dien-1-ol	0.4
geranic acid	1.6
geranyl acetate	1.0
limonene	59.2
limonene glycol	2.9
limonene oxide, trans-	0.7
myrtenal	0.2
nerol acetate	0.9
o-cymene	7.9
sabinene	1.0
α-citral	7.8
α-pinene	1.1
β-citral	3.3
β-myrcene	0.7
β-pinene	7.2
γ-terpinene	1.1

	EO36
artemisia alcohol	1.7
borneol	0.8
borneol, butyrate	1.7
bornyl acetate	1.0
camphene	0.4
β-caryophyllene	6.0
caryophyllene oxide	0.7
cis-3-pinanone	0.3
eucalyptol	2.5
germacrene d	1.8
limonene	7.3
o-cymene	0.2
pinocarveol	4.8
pinocarvone	0.3
sabinene	0.4
sativene	0.7
spathulenol	1.3
terpinen-4-ol	0.3
terpinolene	0.2
trans-2,7-dimethyl-4,6-octadien-2-ol	49.3
yomogi alcohol	1.3
α-pinene	18.9
β-myrcene	1.7
δ-elemene	1.5

	EO37
1-octen-3-ol	0.9
borneol	1.0
carvacrol	71.5
carvacrol acetate	0.3
β-caryophyllene	5.0
caryophyllene oxide	1.0
eucalyptol	0.4
limonene	0.2
linalool	1.5
p-cymene	5.6
spathulenol	0.3
terpinen-4-ol	1.1
thymol	1.6
α-pinene	0.3
α-terpinene	0.5
α-terpineol	0.2
β-bisabolene	4.1
β-myrcene	0.3
γ-cadinene	0.4
γ-muurolene	0.6
γ-terpinene	2.3
δ-cadinene	0.8

	EO38
borneol	1.3
bornyl acetate	0.4
camphene	0.8
camphor	21.4
β-caryophyllene	6.2
eucalyptol	53.2
eugenol	0.7
limonene	1.7
o-cymene	0.4
terpinen-4-ol	0.4
α-pinene	6.7
α-terpineol	1.2
β-myrcene	0.3
β-pinene	5.3

	EO39
2-methoxycinnamaldehyde	0.2
acetic acid, cinnamyl ester	2.6
acetyleugenol	1.3
β-caryophyllene	4.0
caryophyllene oxide	0.2
eugenol	34.6
humulene	0.6
limonene	0.2
linalool	3.2
o-cymene	0.7
tetradecanal	0.5
trans-3-phenyl-2-propenal	49.1
α-copaene	0.5
α-phellandrene	0.3
α-pinene	0.2
α-terpineol	0.3
β-isosafrole	0.9
β-phellandrene	0.5

	EO40
carvone	0.5
cis-carveol	0.2
eucalyptol	85.9
limonene	4.5
o-cymene	8.3
α-pinene	0.6

	EO41
carvone	0.8
cis-carveol	0.5
cis-p-mentha-2,8-dien-1-ol	0.3
limonene	95.0
linalool	0.8
sabinene	0.2
trans-p-mentha-2,8-dienol	0.3
α-pinene	0.4
β-myrcene	1.7

	EO42
β-caryophyllene	1.8
eucalyptol	66.0
ledol	5.4
limonene	5.9
linalool	0.2
o-cymene	1.5
terpinen-4-ol	0.9
terpinolene	0.4
α-pinene	5.3
α-terpineol	8.3
α-terpineol acetate	1.4
β-myrcene	0.7
β-pinene	1.5
γ-terpinene	0.8

	EO43
artemisia alcohol	2.0
borneol	0.9
camphene	3.0
camphor	27.0
cumaldehyde	0.4
eucalyptol	2.0
germacrene D	0.3
myrtenol	0.3
o-cymene	1.2
pinocarveol	0.7
sabinene	0.9
santolina triene	2.2
terpinen-4-ol	2.5
thujone	44.6
yomogi alcohol	1.6
α-pinene	0.2
β-thujone	9.9
γ-terpinene	0.3

	EO44
β-caryophyllene	9.1
cis-β-terpineol	0.7
eucalyptol	52.7
limonene	3.6
linalool	2.2
p-cymene	18.4
p-menth-3-en-1-ol	0.3
terpinolene	0.5
α-pinene	1.5
α-terpineol	7.6
α-terpineol acetate	1.2
β-myrcene	0.6
β-pinene	0.8
γ-terpinene	0.9

	EO45
3-carene	5.9
calamenene	0.3
β-caryophyllene	33.6
caryophyllene oxide	9.7
humulene	2.4
humulene epoxide 2	0.5
limonene	11.1
linalool	0.6
p-cymene	1.1
sabinene	7.2
spathulenol	0.4
terpinen-4-ol	0.4
α-bergamotene	0.1
α-copaene	5.4
α-muurolene	0.4
α-pinene	6.9
β-bisabolene	2.0
β-cubebene	0.7
β-elemene	0.7
β-pinene	6.3
β-selinene	0.5
γ-muurolene	0.2
δ-cadinene	0.9
δ-elemene	2.7

	EO46
1-octen-3-ol	0.5
borneol	2.2
camphene	1.0
camphor	2.4
carvacrol	6.6
β-caryophyllene	2.4
caryophyllene oxide	0.7
cis-β-terpineol	0.2
eucalyptol	1.2
limonene	0.5
linalool	7.5
methyl thymyl ether	0.4
p-cymene	18.2
terpinen-4-ol	3.0
thymol	44.4
α-pinene	1.3
α-terpinene	0.6
α-terpineol	0.2
β-myrcene	1.1
β-pinene	0.3
γ-terpinene	5.2

	EO47
1,4-dihydroxy-p-menth-2-ene	0.8
β-caryophyllene	0.6
caryophyllene oxide	1.0
cis-sabinene hydrate	2.9
cis-β-terpineol	15.4
eucalyptol	1.0
limonene	0.8
linalol oxide	0.5
linalool	40.7
o-cymene	8.5
p-cymen-8-ol	0.6
sabinene	2.6
terpinen-4-ol	23.2
α-pinene	0.4
α-terpineol	0.4
β-myrcene	0.5

	EO48
acetyleugenol	9.9
β-caryophyllene	8.8
eugenol	80.1
humulene	1.0
methylsalicylate	0.2

	EO49
3-carene	29.3
camphene	0.6
cedrene	0.5
cedrol	4.7
cis-verbenol	0.3
eucalyptol	0.5
limonene	4.0
linalool	0.7
o-cymene	1.4
p-cymen-8-ol	1.2
sabinene	0.7
terpinen-4-ol	1.5
terpinolene	0.7
trans-2-caren-4-ol	0.4
verbenone	0.3
α-pinene	47.7
α-terpineol	0.4
α-terpineol acetate	3.8
β-pinene	1.3

	EO50
eucalyptol	2.9
eugenol	2.2
geranyl acetate	0.2
isoeugenol	0.3
limonene	7.8
linalool	0.5
methyleugenol	0.3
myristicin	21.9
o-cymene	1.6
sabinene	13.9
terpinen-4-ol	16.6
terpinolene	0.7
α-copaene	0.3
α-phellandrene	0.8
α-pinene	12.0
α-terpinene	0.6
α-terpineol	0.7
α-terpineol acetate	0.1
β-isosafrole	1.5
β-pinene	11.1
γ-terpinene	3.8

	EO51
β-caryophyllene	0.2
eucalyptol	0.9
isomenthone	10.0
isopulegol	0.9
levomenthol	0.4
limonene	0.5
menthol	57.4
menthol, acetate	5.0
menthone	23.2
piperitenone	0.7
pulegone	0.4
α-terpineol	0.3

	EO52
6-methyl-5-hepten-2-one	1.3
β-caryophyllene	0.7
cis-geraniol	0.6
cis-verbenol	0.3
citronellal	8.0
citronellyl formate	1.1
elemol	0.5
eucalyptol	0.6
geranic acid	0.7
geraniol	5.6
limonene	9.5
linalool	1.3
sabinene	0.5
terpinen-4-ol	0.2
α-citral	33.7
α-copaene	0.1
α-pinene	1.6
β-citral	30.9
β-myrcene	0.4
β-pinene	2.1
δ-cadinene	0.2

	EO53
β-caryophyllene	0.3
caryophyllene oxide	0.3
cis-α-bisabolene	2.1
cis-β-farnesene	0.3
estragole	75.2
levomenthol	0.4
linalool	19.7
α-bergamotene	0.9
α-citral	0.7

	EO54
carvone	0.6
β-caryophyllene	1.8
cis-geraniol	76.5
cis-β-ocimene	0.3
farnesol	0.7
geranyl acetate	13.0
humulene	0.2
linalool	4.1
nerol acetate	1.0
α-citral	0.7
β-ocimene	1.1

	EO55
eucalyptol	53.9
eugenol	1.9
limonene	2.0
linalool	3.0
methyleugenol	4.2
myrtenol	0.3
o-cymene	2.4
sabinene	3.2
terpinen-4-ol	3.0
α-pinene	3.2
α-terpineol acetate	18.8
β-pinene	2.3
γ-terpinene	1.8

	EO56
anethole	88.5
β-caryophyllene	0.4
estragole	3.8
isohomogenol	0.2
limonene	1.6
linalool	0.9
nerolidol	0.3
p-acetonylanisole	0.9
p-anisaldehyde	2.1
terpinen-4-ol	0.3
α-bergamotene	0.5
α-pinene	0.3
α-terpineol	0.2

	EO57
3-carene	0.5
4-epi-cubebol	1.7
acetic acid	0.7
bornyl acetate	1.0
β-caryophyllene	9.1
caryophyllene oxide	3.0
cis-verbenol	1.8
humulene	2.0
humulene epoxide 2	1.1
limonene	20.3
myrtenyl acetate	0.3
o-cymene	3.7
p-cymen-8-ol	0.2
pinocarveol	0.9
sabinene	2.8
terpinen-4-ol	0.6
viridiflorol	0.5
α-copaene	2.3
α-cubebene	0.4
α-muurolene	0.4
α-phellandrene	2.0
α-pinene	27.0
α-selinene	1.1
α-terpineol	0.5
β-elemene	4.0
β-eudesmol	0.3
β-myrcene	3.0
β-phellandrene	1.0
β-pinene	1.0
β-selinene	1.6
γ-eudesmol	0.5
δ-cadinene	2.1
τ-cadinol	2.3

	EO58
.τ.-muurolol	0.1
calamenene	0.7
β-caryophyllene	7.3
caryophyllene oxide	0.2
cinerolon	4.6
cubenol	0.2
cyclohexanone, 2-(1-methylethylidene)-	1.0
epi-bicyclosesquiphellandrene	0.9
germacrene D	5.2
humulene	1.1
jasmone	0.6
limonene	8.8
myrtenal	0.3
pulegone	59.8
sabinene	0.6
terpinen-4-ol	0.7
thymol	1.2
α-cadinol	0.2
α-cubebene	0.5
α-gurjunene	0.4
α-muurolene	0.2
α-pinene	1.0
β-ocimene	0.1
β-pinene	3.8
γ-cadinene	0.2
γ-terpinene	0.1

	EO59
1-octen-3-ol	0.7
borneol	0.6
carvacrol	61.0
β-caryophyllene	13.6
caryophyllene oxide	1.4
cis-β-terpineol	0.2
humulene	0.6
limonene	0.3
linalool	1.8
p-cymene	7.1
thymol	0.5
α-citral	0.2
α-terpinene	1.5
α-thujene	1.0
β-bisabolene	1.6
β-myrcene	1.6
β-phellandrene	0.3
γ-terpinene	6.0

	EO60
1-octen-3-ol	0.6
borneol	2.5
camphene	0.6
carvacrol	0.7
β-caryophyllene	3.4
caryophyllene oxide	1.8
eucalyptol	0.9
isothymol methyl ether	6.0
limonene	0.4
linalool	3.7
methyl thymyl ether	10.7
p-cymene	30.5
thymol	34.1
α-pinene	0.9
α-terpineol	0.3
β-myrcene	1.0
γ-muurolene	0.3
γ-terpinene	1.8

	EO61
1-octen-3-ol	0.5
alcanfor	0.3
borneol	0.3
carvacrol	0.8
β-caryophyllene	2.9
caryophyllene oxide	0.6
eucalyptol	0.5
humulene	0.3
isothymol methyl ether	7.4
limonene	0.4
methyl thymyl ether	4.9
p-cymen-8-ol	0.1
p-cymene	18.5
thymol	34.7
thymol acetate	0.3
α-muurolene	0.2
α-phellandrene	0.2
α-terpineol	0.2
α-thujene	1.1
β-bisabolene	5.0
β-bourbonene	0.4
β-myrcene	1.3
γ-cadinene	0.4
γ-muurolene	0.6
γ-terpinene	17.1
δ-cadinene	0.7

Table S10. Preliminary models developed with the procedure described in reference [5].

Threshold	Strain	ML Method	MCC_{CV}
40	39P	svm	0.485
40	39P	lr	0.426
120	27P	dt	0.485
120	39P	rf	0.429
120	39P	svm	0.612
120	39P	svm	0.612
120	39P	svm	0.477
120	39P	lr	0.492
120	39P	knn	0.477

Table S11. Preliminary models developed for threshols at 80% and 100% biofilm modulation [5].

Threshold	Strain	ML Method	MCC_{CV}
80	PaO1	dt	0.408
80	39P	rf	0.426
80	39P	rf	0.418
80	39P	gb	0.566
80	39P	gb	0.418
80	39P	lr	0.473
80	39P	lr	0.473
80	39P	lr	0.443
80	39P	lr	0.41
80	39P	dt	0.426
80	39P	knn	0.497
100	39P	svm	0.405
100	39P	dt	0.539
100	39P	knn	0.585

Table S12. Number of model evaluated during the ML optimization process. NA means no model were developed for the strain/threshold combination due to to low number of active or inactive samples.

Type	Iters	T	PA14	PaO1	22P	25P	26P	27P	37P	39P	Sum	3 Best for each
Coarse	10	40	2880	NA	2880	2880	NA	2880	2880	2880	17280	17298
Finer	100		16000	NA	8000	16000	NA	16000	24000	8000	88000	88018
Extra	1000		1000	NA	1000	1000	NA	1000	1000	1000	6000	6018
Final	10000		10000	NA	10000	10000	NA	10000	10000	10000	60000	60018
Coarse	10	120	NA	2880	NA	2880	2880	2880	NA	2880	14400	14415
Finer	100		NA	8000	NA	16000	24000	16000	NA	16000	80000	80015
Extra	1000		NA	1000	NA	1000	1000	1000	NA	1000	5000	5015
Final	10000		NA	10000	NA	10000	10000	10000	NA	10000	50000	50015
Sum			29880	21880	21880	59760	37880	59760	37880	51760	320680	320812

Table S13. Preliminary models P1-P33 obtained with the combination of DA and randome search hyperparamenter optimization.

# Model	threshold	strain	MCC		ACC		ML	comp min	Preprocess	pc
			cv	fit	cv	fit	Method		function	
**P1**	40	PA14	0.46	1.00	0.87	1.00	gb	2	baug20	0.6
**P2**		PA14	0.44	0.65	0.83	0.89	rf	0	baug20	0.8
**P3**		PA14	0.41	1.00	0.87	1.00	dt	2	baug10	0.8
**P4**		22P	0.47	0.47	0.78	0.78	dt	0	aug20	0
**P5**		22P	0.47	0.47	0.78	0.78	dt	0	aug20	0
**P6**		22P	0.47	0.47	0.78	0.78	dt	0	baug20	0
**P7**		25P	0.39	0.96	0.79	0.98	dt	2	baug10	0.8
**P8**		25P	0.36	0.88	0.71	0.95	gb	6	baug10	0.6
**P9**		25P	0.34	1.00	0.75	1.00	gb	2	aug20	0.8
**P10**		27P	0.57	0.93	0.89	0.98	gb	6	aug10	0.6
**P11**		27P	0.55	1.00	0.89	1.00	rf	6	aug10	0.6
**P12**		27P	0.55	1.00	0.89	1.00	gb	6	aug10	0.8
**P13**		37P	0.48	0.78	0.78	0.90	rf	0	baug20	0.8
**P14**		37P	0.43	0.60	0.78	0.84	knn	6	aug10	0.6
**P15**		37P	0.42	0.56	0.76	0.80	gb	6	baug10	0.6
**P16**		39P	0.52	0.60	0.76	0.80	rf	0	aug20	0.8
**P17**		39P	0.52	0.56	0.76	0.78	rf	6	aug10	0.8
**P18**		39P	0.51	0.63	0.76	0.82	rf	6	aug10	0.8
**P19**	120	PaO1	0.52	0.32	0.77	0.68	svm	6	baug20	0.8
**P20**		PaO1	0.50	0.22	0.75	0.63	svm	6	baug10	0.8
**P21**		PaO1	0.48	0.71	0.74	0.86	svm	6	baug10	1.0
**P22**		25P	0.47	0.47	0.71	0.71	dt	0	baug20	0.8
**P23**		25P	0.41	0.71	0.82	0.89	svm	6	baug10	1.0
**P24**		25P	0.40	0.25	0.71	0.41	dt	0	baug10	1.0
**P25**		26P	0.59	0.81	0.82	0.91	rf	0	baug10	0.8
**P26**		26P	0.57	0.78	0.82	0.89	dt	2	baug20	0.8
**P27**		26P	0.56	0.76	0.81	0.89	rf	0	aug20	0.8
**P28**		27P	0.47	1.00	0.77	1.00	gb	2	baug10	0.8
**P29**		27P	0.46	0.60	0.77	0.82	svm	0	aug10	1.0
**P30**		27P	0.46	0.60	0.77	0.82	svm	0	aug20	0.8
**P31**		39P	0.78	1.00	0.96	1.00	gb	2	baug10	0.6
**P32**		39P	0.78	1.00	0.96	1.00	dt	2	baug10	0.6
**P33**		39P	0.76	1.00	0.96	1.00	dt	0	aug10	0.6

Table S14. Preliminary models P1-P33 associated hyperparamenters

# Models	Hyperparameters
**P1**	{'n_estimators': 191, 'max_depth': 11, 'min_samples_leaf': 1}
**P2**	{'class_weight': {0: 1.0, 1: 1.0}, 'n_estimators': 51, 'max_depth': 121, 'min_samples_leaf': 191}
**P3**	{'class_weight': {0: 1.0, 1: 1.1}, 'criterion': 'gini', 'splitter': 'best', 'max_depth': 11, 'min_samples_split': 16, 'min_samples_leaf': 11, 'max_features': None}
**P4**	{'class_weight': {0: 3.0, 1: 1.0}, 'criterion': 'gini', 'splitter': 'best', 'max_depth': 1, 'min_samples_split': 6, 'min_samples_leaf': 6, 'max_features': None}
**P5**	{'class_weight': {0: 1.0, 1: 1.4}, 'criterion': 'gini', 'splitter': 'best', 'max_depth': 6, 'min_samples_split': 1, 'min_samples_leaf': 16, 'max_features': 'auto'}
**P6**	{'class_weight': {0: 1.0, 1: 1.1}, 'criterion': 'entropy', 'splitter': 'best', 'max_depth': 1, 'min_samples_split': 16, 'min_samples_leaf': 11, 'max_features': None}
**P7**	{'class_weight': {0: 1.0, 1: 1.1}, 'criterion': 'gini', 'splitter': 'best', 'max_depth': 11, 'min_samples_split': 11, 'min_samples_leaf': 16, 'max_features': 'auto'}
**P8**	{'n_estimators': 191, 'max_depth': 191, 'min_samples_leaf': 121}
**P9**	{'n_estimators': 81, 'max_depth': 181, 'min_samples_leaf': 1}
**P10**	{'n_estimators': 81, 'max_depth': 171, 'min_samples_leaf': 41}
**P11**	{'class_weight': {0: 1.4, 1: 1.0}, 'n_estimators': 51, 'max_depth': 41, 'min_samples_leaf': 1}
**P12**	{'n_estimators': 21, 'max_depth': 31, 'min_samples_leaf': 11}
**P13**	{'class_weight': {0: 1.0, 1: 1.1}, 'n_estimators': 171, 'max_depth': 91, 'min_samples_leaf': 161}
**P14**	{'n_neighbors': 26, 'weights': 'uniform', 'algorithm': 'brute', 'leaf_size': 11, 'metric': 'minkowski', 'metric_params': None, 'p': 1}
**P15**	{'n_estimators': 11, 'max_depth': 161, 'min_samples_leaf': 71}
**P16**	{'class_weight': {0: 1.5, 1: 1.0}, 'n_estimators': 151, 'max_depth': 171, 'min_samples_leaf': 181}
**P17**	{'class_weight': {0: 1.0, 1: 1.1}, 'n_estimators': 111, 'max_depth': 131, 'min_samples_leaf': 111}
**P18**	{'class_weight': {0: 1.0, 1: 1.0}, 'n_estimators': 31, 'max_depth': 91, 'min_samples_leaf': 61}
**P19**	{'class_weight': {0: 1.0, 1: 1.3}, 'C': 0.3418, 'kernel': 'sigmoid', 'probability': True}
**P20**	{'class_weight': {0: 1.0, 1: 1.5}, 'C': 21.6, 'kernel': 'sigmoid', 'probability': True}
**P21**	{'class_weight': {0: 1.2, 1: 1.0}, 'C': 8.3, 'kernel': 'linear', 'probability': True}
**P22**	{'class_weight': {0: 1.4, 1: 1.0}, 'criterion': 'entropy', 'splitter': 'best', 'max_depth': 1, 'min_samples_split': 11, 'min_samples_leaf': 11, 'max_features': None}
**P23**	{'class_weight': {0: 2.5, 1: 1.0}, 'C': 1.4, 'kernel': 'rbf', 'probability': True}
**P24**	{'class_weight': {0: 1.0, 1: 1.2}, 'criterion': 'entropy', 'splitter': 'random', 'max_depth': 1, 'min_samples_split': 16, 'min_samples_leaf': 1, 'max_features': None}
**P25**	{'class_weight': {0: 1.3, 1: 1.0}, 'n_estimators': 161, 'max_depth': 181, 'min_samples_leaf': 41}
**P26**	{'class_weight': {0: 1.0, 1: 1.1}, 'criterion': 'gini', 'splitter': 'random', 'max_depth': 11, 'min_samples_split': 6, 'min_samples_leaf': 6, 'max_features': 'auto'}
**P27**	{'class_weight': {0: 3.0, 1: 1.0}, 'n_estimators': 191, 'max_depth': 151, 'min_samples_leaf': 81}
**P28**	{'n_estimators': 91, 'max_depth': 141, 'min_samples_leaf': 71}
**P29**	{'class_weight': {0: 1.5, 1: 1.0}, 'C': 0.0954, 'kernel': 'sigmoid', 'probability': True}
**P30**	{'class_weight': {0: 1.0, 1: 1.0}, 'C': 1.6, 'kernel': 'linear', 'probability': True}
**P31**	{'n_estimators': 111, 'max_depth': 81, 'min_samples_leaf': 51}

**P32**	{'class_weight': {0: 1.0, 1: 3.0}, 'criterion': 'entropy', 'splitter': 'best', 'max_depth': 16, 'min_samples_split': 11, 'min_samples_leaf': 6, 'max_features': None}
**P33**	{'class_weight': {0: 1.4, 1: 1.0}, 'criterion': 'gini', 'splitter': 'best', 'max_depth': 6, 'min_samples_split': 16, 'min_samples_leaf': 1, 'max_features': 'sqrt'}

Table S15. Intermediate ML models I100_1-I100_11 with the data augmentation and 100 random iterations

# Model	threshold	strain	MCC		ACC		ML	comp min	Preprocess	pc
			cv	fit	cv	fit	Method		function	
**I100_1**	40	PA14	0.68	1.00	0.93	1.00	gb	0	baug10	0.8
**I100_2**		22P	0.47	0.47	0.78	0.78	dt	2	aug10	1.0
**I100_3**		25P	0.52	0.95	0.82	0.98	gb	4	aug20	0.8
**I100_4**		27P	0.72	1.00	0.93	1.00	gb	2	aug10	0.8
**I100_5**		37P	0.71	0.91	0.88	0.96	gb	0	baug10	0.9
**I100_6**		39P	0.64	1.00	0.82	1.00	rf	4	aug20	0.9
**I100_7**	120	PaO1	0.57	0.27	0.79	0.65	svm	6	aug10	0.8
**I100_8**		25P	0.71	0.67	0.89	0.88	dt	0	baug20	0.9
**I100_9**		26P	0.73	1.00	0.89	1.00	dt	2	baug20	0.9
**I100_10**		27P	0.64	0.26	0.84	0.70	svm	0	aug20	0.8
**I100_11**		39P	0.88	1.00	0.98	1.00	gb	2	baug10	0.6

Table S16. Intermediate ML models I100_1-I100_11 associated hyperparamenters as listed in Table SM15

# Models	Hyperparameters
**I100_1**	{'n_estimators': 189, 'max_depth': 3, 'min_samples_leaf': 160}
**I100_2**	{'class_weight': {0: 1.5, 1: 1.0}, 'criterion': 'gini', 'splitter': 'best', 'max_depth': 1, 'min_samples_split': 11, 'min_samples_leaf': 2, 'max_features': None}
**I100_3**	{'n_estimators': 27, 'max_depth': 140, 'min_samples_leaf': 153}
**I100_4**	{'n_estimators': 38, 'max_depth': 162, 'min_samples_leaf': 52}
**I100_5**	{'n_estimators': 71, 'max_depth': 47, 'min_samples_leaf': 199}
**I100_6**	{'class_weight': {0: 1.5, 1: 1.0}, 'n_estimators': 98, 'max_depth': 124, 'min_samples_leaf': 5}
**I100_7**	{'class_weight': {0: 1.5, 1: 1.0}, 'C': 1.0207, 'kernel': 'sigmoid', 'probability': True}
**I100_8**	{'class_weight': {0: 1.0, 1: 1.2}, 'criterion': 'entropy', 'splitter': 'best', 'max_depth': 2, 'min_samples_split': 6, 'min_samples_leaf': 14, 'max_features': None}
**I100_9**	{'class_weight': {0: 1.0, 1: 1.1}, 'criterion': 'gini', 'splitter': 'best', 'max_depth': 6, 'min_samples_split': 13, 'min_samples_leaf': 12, 'max_features': 'sqrt'}
**I100_10**	{'class_weight': {0: 1.0, 1: 3.0}, 'C': 21.6, 'kernel': 'sigmoid', 'probability': True}
**I100_11**	{'n_estimators': 99, 'max_depth': 76, 'min_samples_leaf': 20}

Table S17. Intermediate ML models with the data augmentation setting selected from models I100_1-I100_11 and 1000 random iterations

# Model	threshold	strain	MCC		ACC		ML Method	comp min	Preprocess function	pc
			cv	fit	cv	fit				
**I1000_1**	40	PA14	0.62	1.00	0.91	1.00	gb	0	baug10	0.8
**I1000_2**		22P	0.47	0.62	0.78	0.83	dt	2	aug10	1.0
**I1000_3**		25P	0.52	0.67	0.82	0.88	gb	4	aug20	0.8
**I1000_4**		27P	0.72	1.00	0.93	1.00	gb	2	aug10	0.8
**I1000_5**		37P	0.71	0.82	0.88	0.92	gb	0	baug10	0.9
**I1000_6**		39P	0.59	0.92	0.80	0.96	rf	4	aug20	0.9
**I1000_7**	120	PaO1	0.67	0.27	0.84	0.65	svm	6	aug10	0.8
**I1000_8**		25P	0.74	0.67	0.91	0.88	dt	0	baug20	0.9
**I1000_9**		26P	0.73	0.96	0.89	0.98	dt	2	baug20	0.9
**I1000_10**		27P	0.64	0.31	0.84	0.72	svm	0	aug20	0.8
**I1000_11**		39P	0.88	1.00	0.98	1.00	gb	2	baug10	0.6

Table S18. Intermediate models I1000_1-I1000_11 associated hyperparamenters as listed in Table SM17

# Models	Hyperparameters
**I1000_1**	{'n_estimators': 161, 'max_depth': 177, 'min_samples_leaf': 160}
**I1000_2**	{'class_weight': {0: 2.5, 1: 1.0}, 'criterion': 'entropy', 'splitter': 'best', 'max_depth': 2, 'min_samples_split': 3, 'min_samples_leaf': 20, 'max_features': None}
**I1000_3**	{'n_estimators': 13, 'max_depth': 40, 'min_samples_leaf': 139}
**I1000_4**	{'n_estimators': 56, 'max_depth': 129, 'min_samples_leaf': 60}
**I1000_5**	{'n_estimators': 30, 'max_depth': 30, 'min_samples_leaf': 131}
**I1000_6**	{'class_weight': {0: 1.0, 1: 1.1}, 'n_estimators': 25, 'max_depth': 183, 'min_samples_leaf': 28}
**I1000_7**	{'class_weight': {0: 1.1, 1: 1.0}, 'C': 21.6, 'kernel': 'sigmoid', 'probability': True}
**I1000_8**	{'class_weight': {0: 2.5, 1: 1.0}, 'criterion': 'gini', 'splitter': 'best', 'max_depth': 2, 'min_samples_split': 8, 'min_samples_leaf': 14, 'max_features': None}
**I1000_9**	{'class_weight': {0: 1.0, 1: 2.5}, 'criterion': 'entropy', 'splitter': 'random', 'max_depth': 11, 'min_samples_split': 8, 'min_samples_leaf': 5, 'max_features': None}
**I1000_10**	{'class_weight': {0: 1.0, 1: 2.5}, 'C': 21.6, 'kernel': 'sigmoid', 'probability': True}
**I1000_11**	{'n_estimators': 63, 'max_depth': 179, 'min_samples_leaf': 47}

Table S19. Final models PF1-PF11 with the data augmentation setting selected from models I1000_1-I1000_11 and 10000 random iterations to seek for the best hyperparameters.

# Model	threshold	strain	MCC		ACC		ML	comp min	Preprocess	pc
			cv	fit	cv	fit	Method		function	
**PF1**	40	PA14	0.68	1.00	0.93	1.00	gb	0	baug10	0.8
**PF2**		22P	0.50	0.70	0.80	0.87	dt	2	aug10	1.0
**PF3**		25P	0.53	1.00	0.82	1.00	gb	4	aug20	0.8
**PF4**		27P	0.79	1.00	0.95	1.00	gb	2	aug10	0.8
**PF5**		37P	0.76	0.76	0.90	0.90	gb	0	baug10	0.9
**PF6**		39P	0.60	1.00	0.80	1.00	rf	4	aug20	0.9
**PF7**	120	PaO1	0.67	0.40	0.84	0.72	svm	6	aug10	0.8
**PF8**		25P	0.74	0.67	0.91	0.88	dt	0	baug20	0.9
**PF9**		26P	0.78	0.96	0.91	0.98	dt	2	baug20	0.9
**PF10**		27P	0.60	0.50	0.82	0.79	svm	0	aug20	0.8
**PF11**		39P	0.88	1.00	0.98	1.00	gb	2	baug10	0.6

aug10: data augmentation function in which the data are increase for 10 times in a range of ±15%,
aug20: data augmentation function in which the data are increase for 20 times in a range of ±15%,
baug10: data augmentation function in which the data are increase for 10 times in a range of ±15% balancing the data among the two classes (active or inactives),
baug20: data augmentation function in which the data are increase for 20 times in a range of ±15% balancing the data among the two classes (active or inactives),
pc: amount of explained variance by principal component (e.g. 0.6 = 60%)
comp min: number of minimal occurrences for a components to be mantained in the calculations

Table S20. Models hyperparamenters as listed in Table SM19

# Models	Hyperparameters
**PF1**	{'n_estimators': 48, 'max_depth': 133, 'min_samples_leaf': 133}
**PF2**	{'class_weight': {0: 2.5, 1: 1.0}, 'criterion': 'gini', 'splitter': 'best', 'max_depth': 3, 'min_samples_split': 12, 'min_samples_leaf': 11, 'max_features': None}
**PF3**	{'n_estimators': 32, 'max_depth': 199, 'min_samples_leaf': 58}
**PF4**	{'n_estimators': 111, 'max_depth': 118, 'min_samples_leaf': 123}
**PF5**	{'n_estimators': 4, 'max_depth': 54, 'min_samples_leaf': 173}
**PF6**	{'class_weight': {0: 1.4, 1: 1.0}, 'n_estimators': 44, 'max_depth': 51, 'min_samples_leaf': 6}
**PF7**	{'class_weight': {0: 1.0, 1: 1.4}, 'C': 10.1, 'kernel': 'sigmoid', 'probability': True}
**PF8**	{'class_weight': {0: 3.0, 1: 1.0}, 'criterion': 'gini', 'splitter': 'best', 'max_depth': 2, 'min_samples_split': 14, 'min_samples_leaf': 1, 'max_features': None}
**PF9**	{'class_weight': {0: 1.0, 1: 1.1}, 'criterion': 'entropy', 'splitter': 'best', 'max_depth': 6, 'min_samples_split': 14, 'min_samples_leaf': 13, 'max_features': None}
**PF10**	{'class_weight': {0: 1.0, 1: 2.5}, 'C': 21.6, 'kernel': 'sigmoid', 'probability': True}
**PF11**	{'n_estimators': 162, 'max_depth': 92, 'min_samples_leaf': 26}

Table S21. Optimized final models obtained with 100 random iterations of data augmentation at threshold values of 80% and 100%.

# Model	Strain	Threshold	ML Method	MCC		ACC		F1	
				Fit	CV	Fit	CV	Fit	CV
**F12**	PaO1	80	svm	0.24	0.36	0.79	0.82	0.33	0.38
**F13**	PA14		svm	0.25	0.58	0.74	0.85	0.83	0.90
**F14**	22P		dt	0.62	0.45	0.78	0.69	0.82	0.75
**F15**	25P		dt	0.95	0.35	0.98	0.77	0.99	0.85
**F16**	26P		rf	0.93	0.34	0.96	0.67	0.95	0.61
**F17**	27P		dt	0.97	0.27	0.98	0.63	0.98	0.62
**F18**	37P		dt	0.92	0.19	0.96	0.59	0.96	0.67
**F19**	39P		gb	1.00	0.68	1.00	0.88	1.00	0.92
**F20**	PaO1	100	dt	0.86	0.25	0.93	0.63	0.92	0.53
**F21**	PA14		dt	0.76	0.76	0.93	0.93	0.96	0.96
**F22**	22P		dt	0.88	0.20	0.96	0.74	0.98	0.84
**F23**	25P		dt	0.95	0.41	0.98	0.79	0.99	0.86
**F24**	26P		gb	1.00	0.66	1.00	0.84	1.00	0.88
**F25**	27P		gb	1.00	0.36	1.00	0.68	1.00	0.71
**F26**	37P		gb	1.00	0.58	1.00	0.90	1.00	0.94
**F27**	39P		gb	1.00	0.58	1.00	0.88	1.00	0.93

Table S22. Occurrences of the EOs' chemical components. Only the most frequent compounds are listed.

Component	#[a]	Component	#[a]	Component	#[a]
limonene	51	p-cymen-8-ol	10	linalyl anthranilate	6
α-pinene	47	terpinolene	9	calamenene	6
β-caryophyllene	39	α-citral	9	β-phellandrene	6
β-myrcene	36	β-bisabolene	9	3-carene	6
linalool	35	carvacrol	8	α-cadinol	6
eucalyptol	33	α-muurolene	8	spathulenol	6
β-pinene	30	γ-muurolene	8	germacrene d	6
o-cymene	29	carvone	8	α-phellandrene	5
α-terpineol	28	bornyl acetate	8	nerol acetate	5
caryophyllene oxide	27	eugenol	7	myrtenol	5
terpinen-4-ol	26	thymol	7	cis-β-farnesene	5
sabinene	22	α-bergamotene	7	γ-cadinene	5
γ-terpinene	21	geranyl acetate	7	estragole	5
borneol	16	camphor	7	cis-β-terpineol	5
humulene	16	pinocarveol	7	cis-verbenol	5
δ-cadinene	13	α-terpinene	7	elemol	4
camphene	13	α-terpineol acetate	7	α-cubebene	4
cis-geraniol	12	1-octen-3-ol	6	cis-carveol	4
p-cymene	11	β-elemene	6	β-citral	4
α-copaene	11	humulene epoxide 2	6	methyl thymyl ether	4

Figure S1. Feature importance for model F1 (see main text Table 1). The top 20 components are displayed.

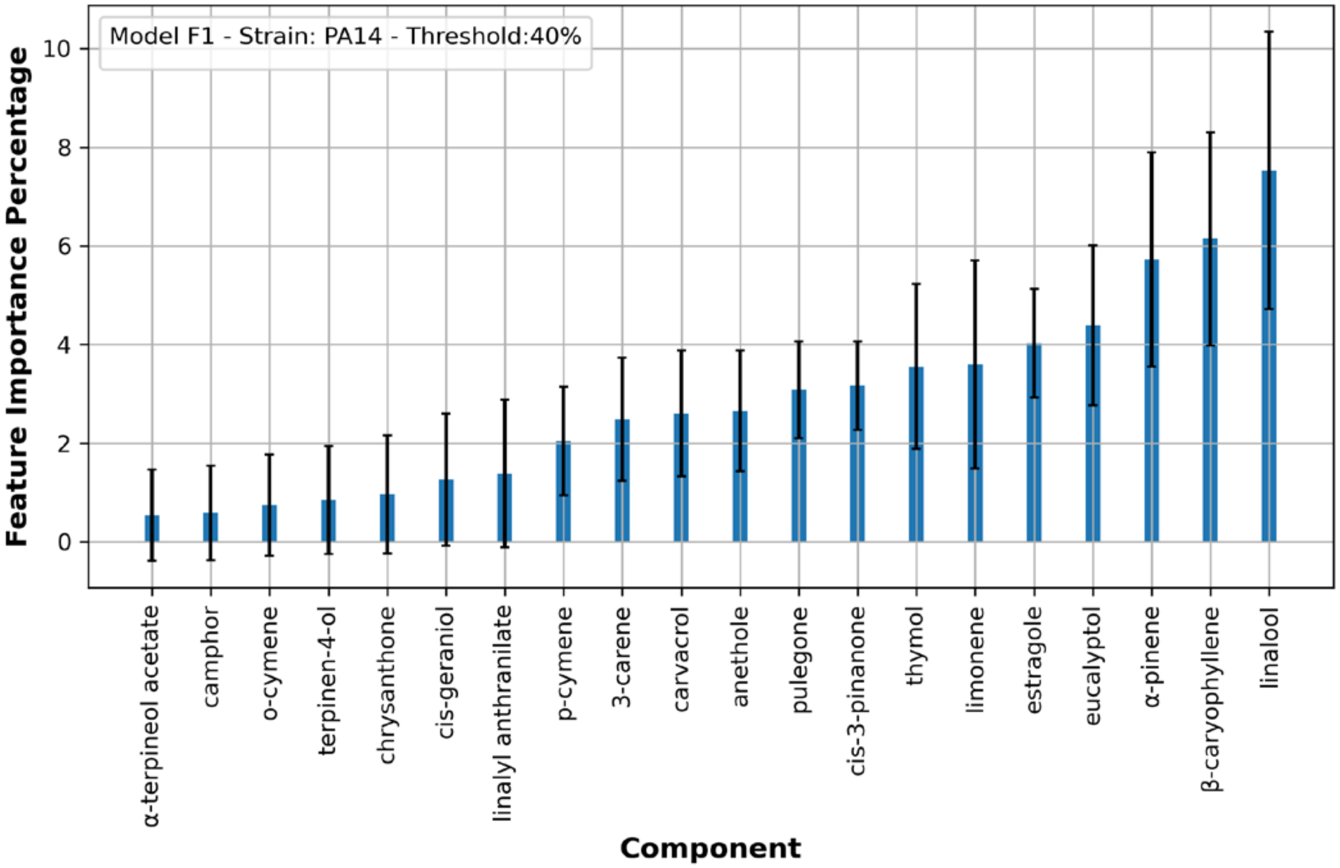

Figure S2. Feature importance for model F2 (see main text Table 1). The top 20 components are displayed.

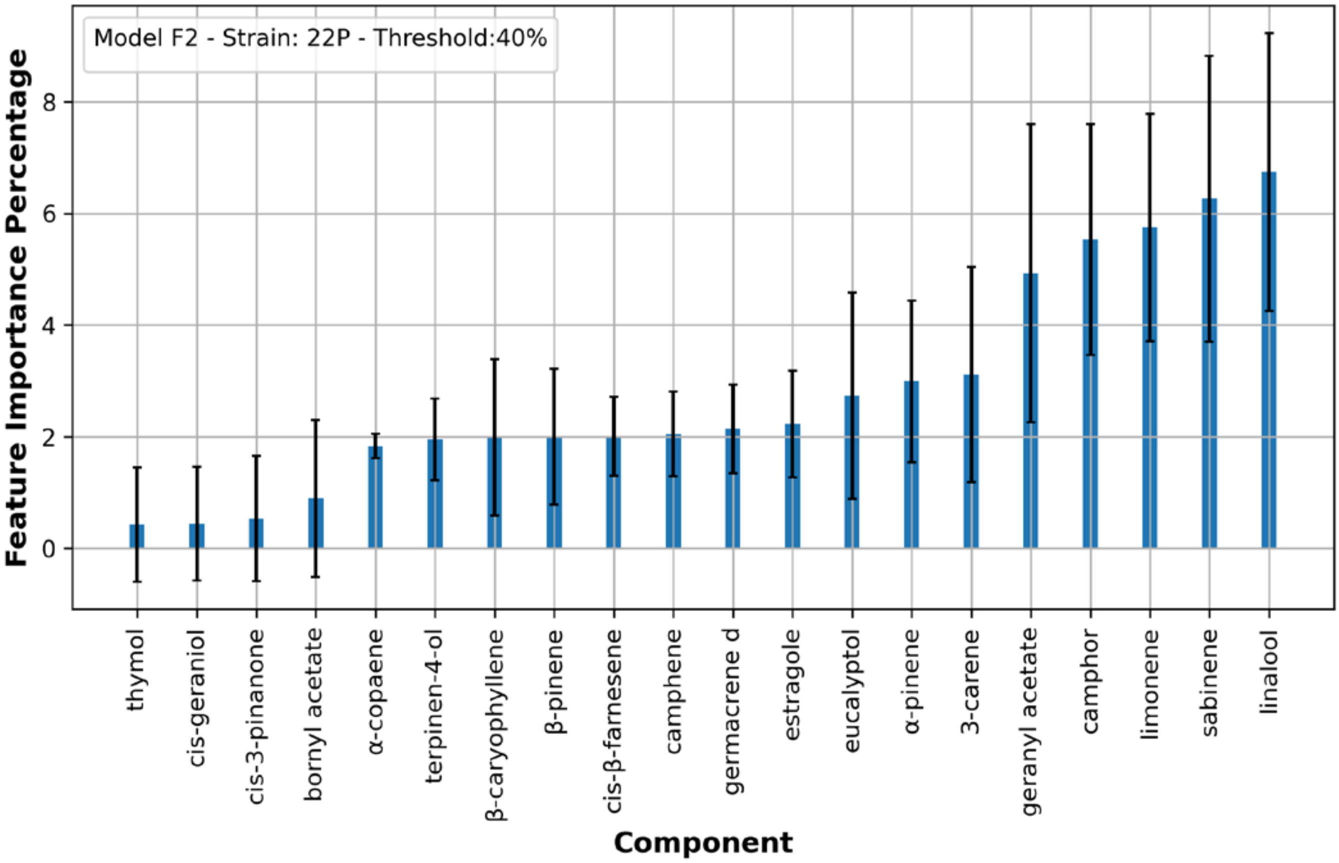

Figure S3. Feature importance for model F3 (see main text Table 1). The top 20 components are displayed.

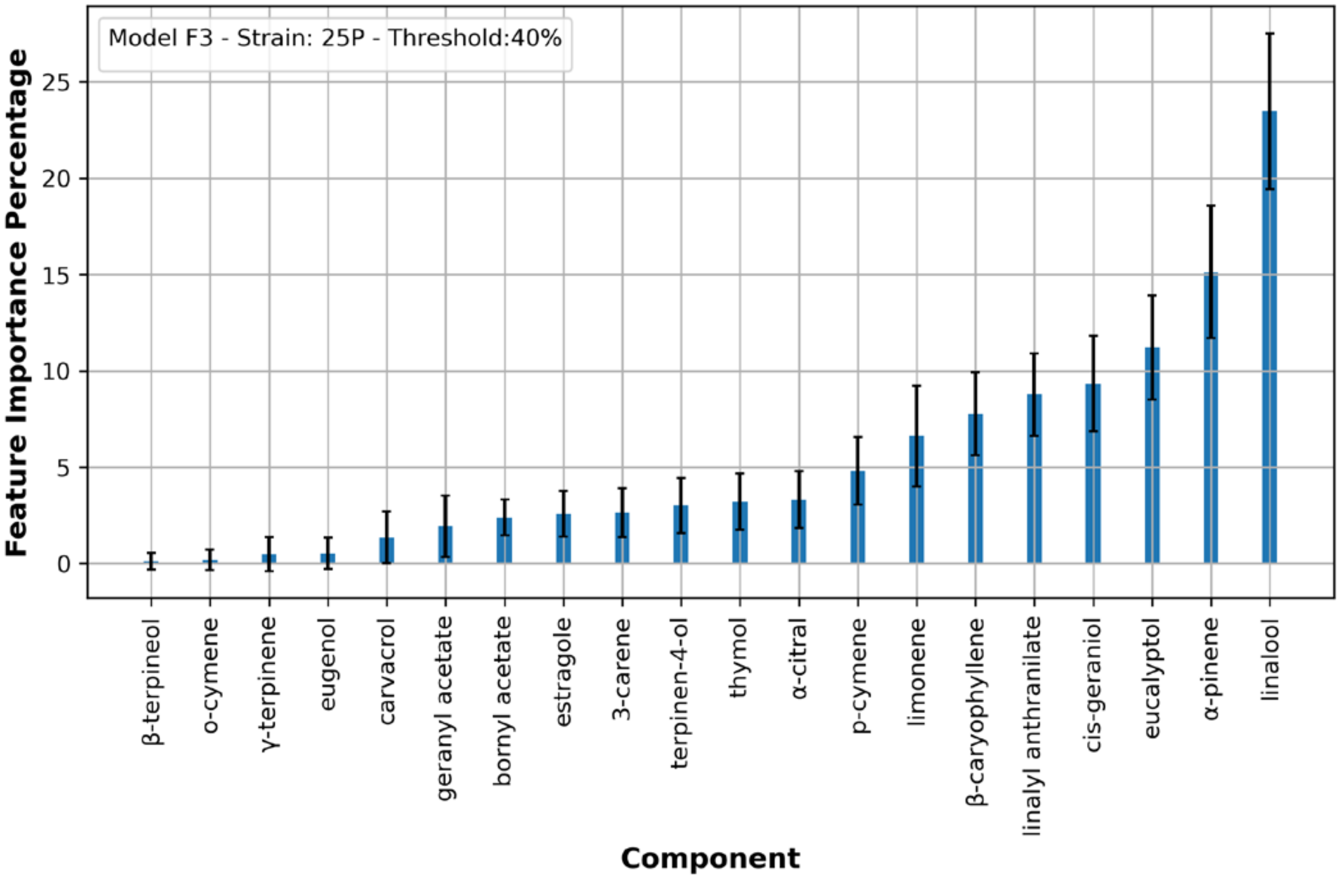

Figure S4. Feature importance for model F4 (see main text Table 1). The top 20 components are displayed.

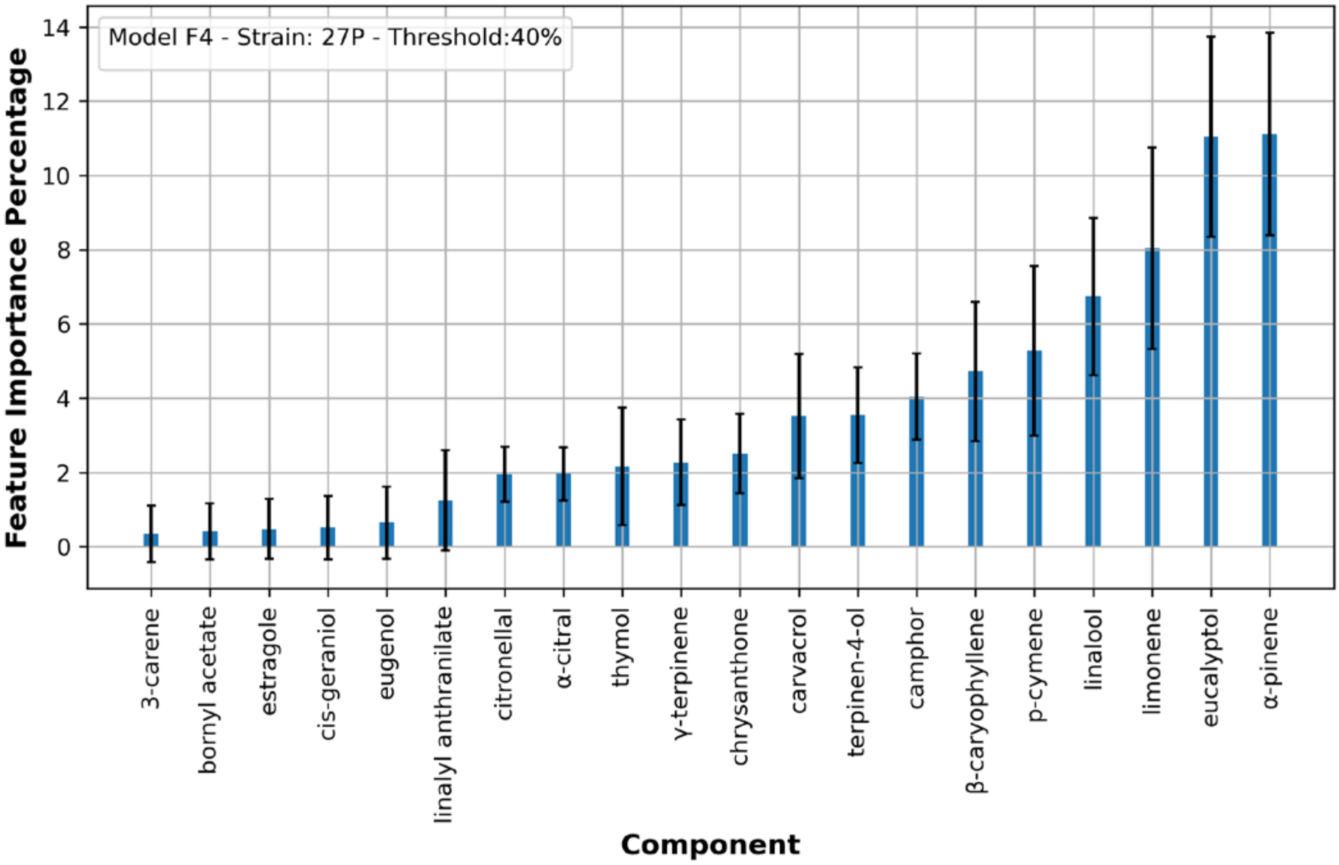

Figure S5. Feature importance for model F5 (see main text Table 1). The top 20 components are displayed.

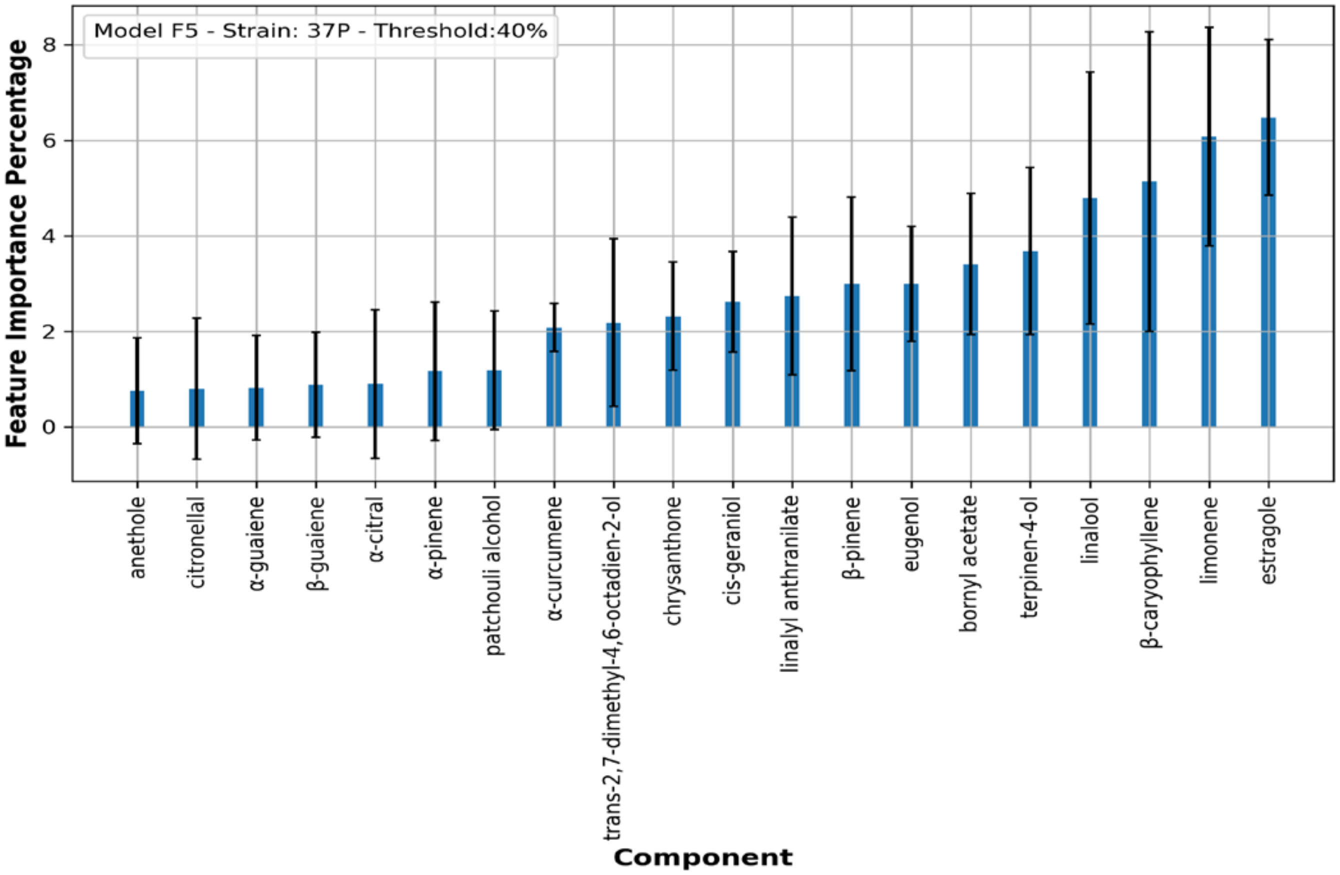

Figure S6. Feature importance for model F6 (see main text Table 1). The top 20 components are displayed.

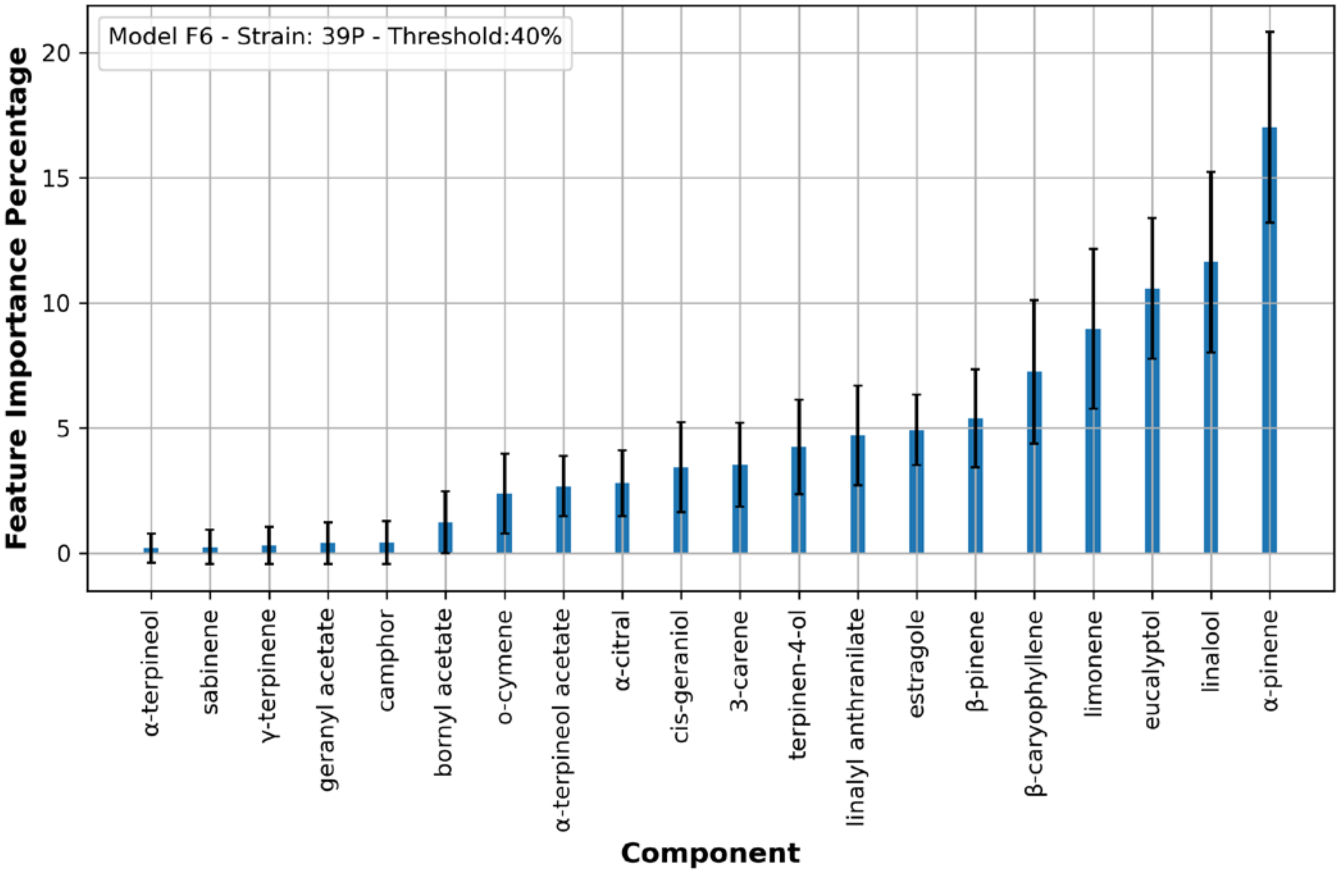

Figure S7. Feature importance for model F7 (see main text Table 1). The top 20 components are displayed.

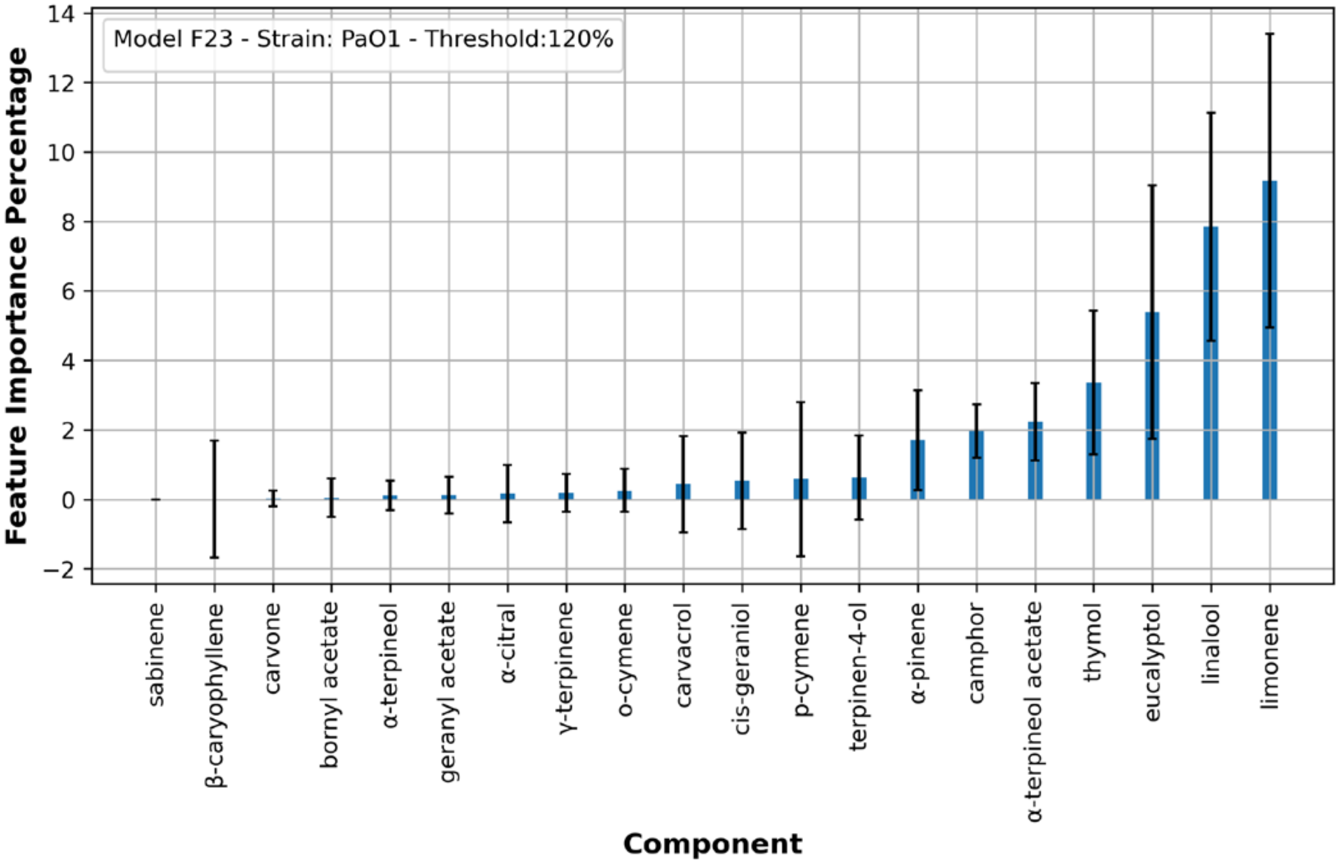

Figure S8. Feature importance for model F8 (see main text Table 1). The top 20 components are displayed.

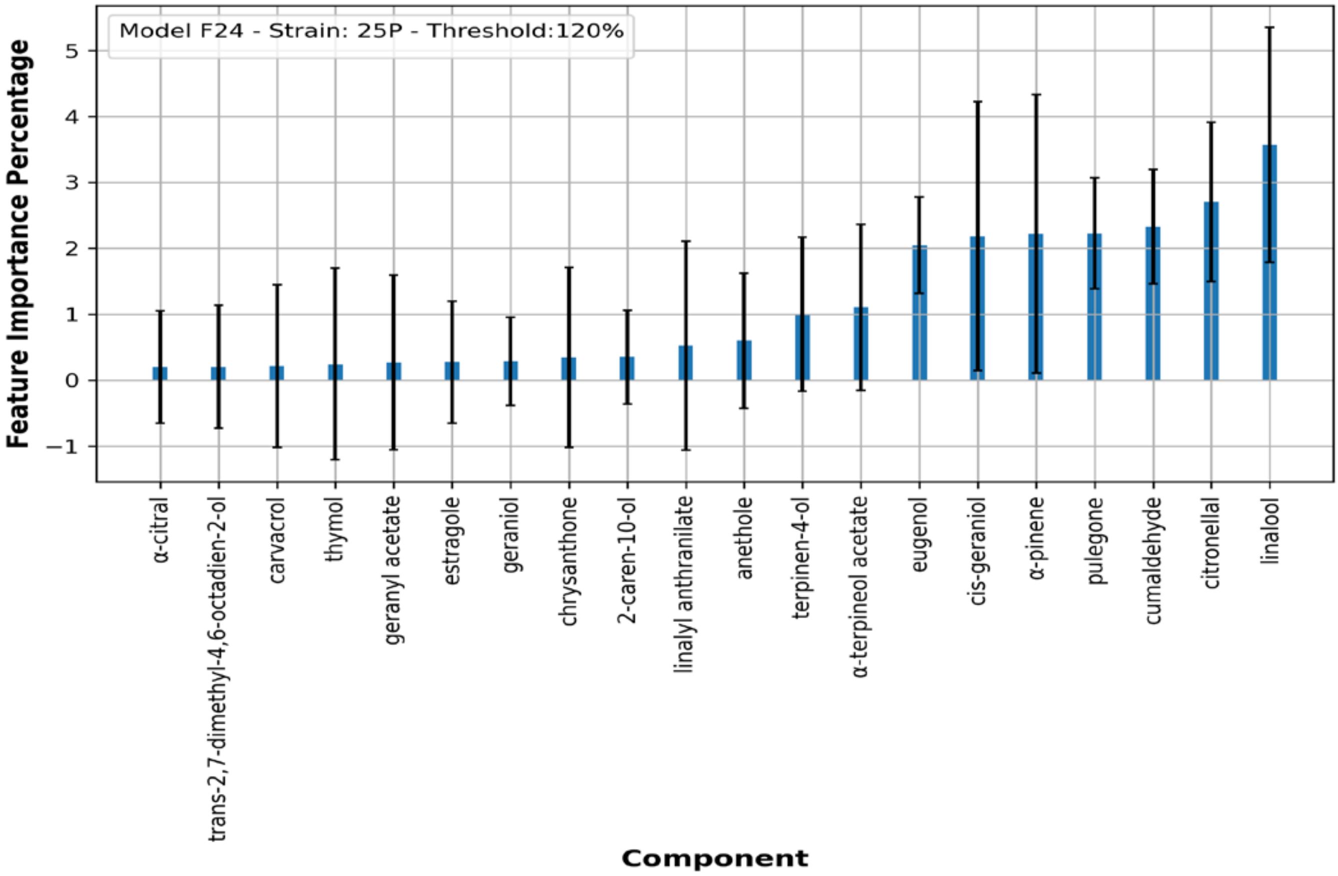

Figure S9. Feature importance for model F9 (see main text Table 1). The top 20 components are displayed.

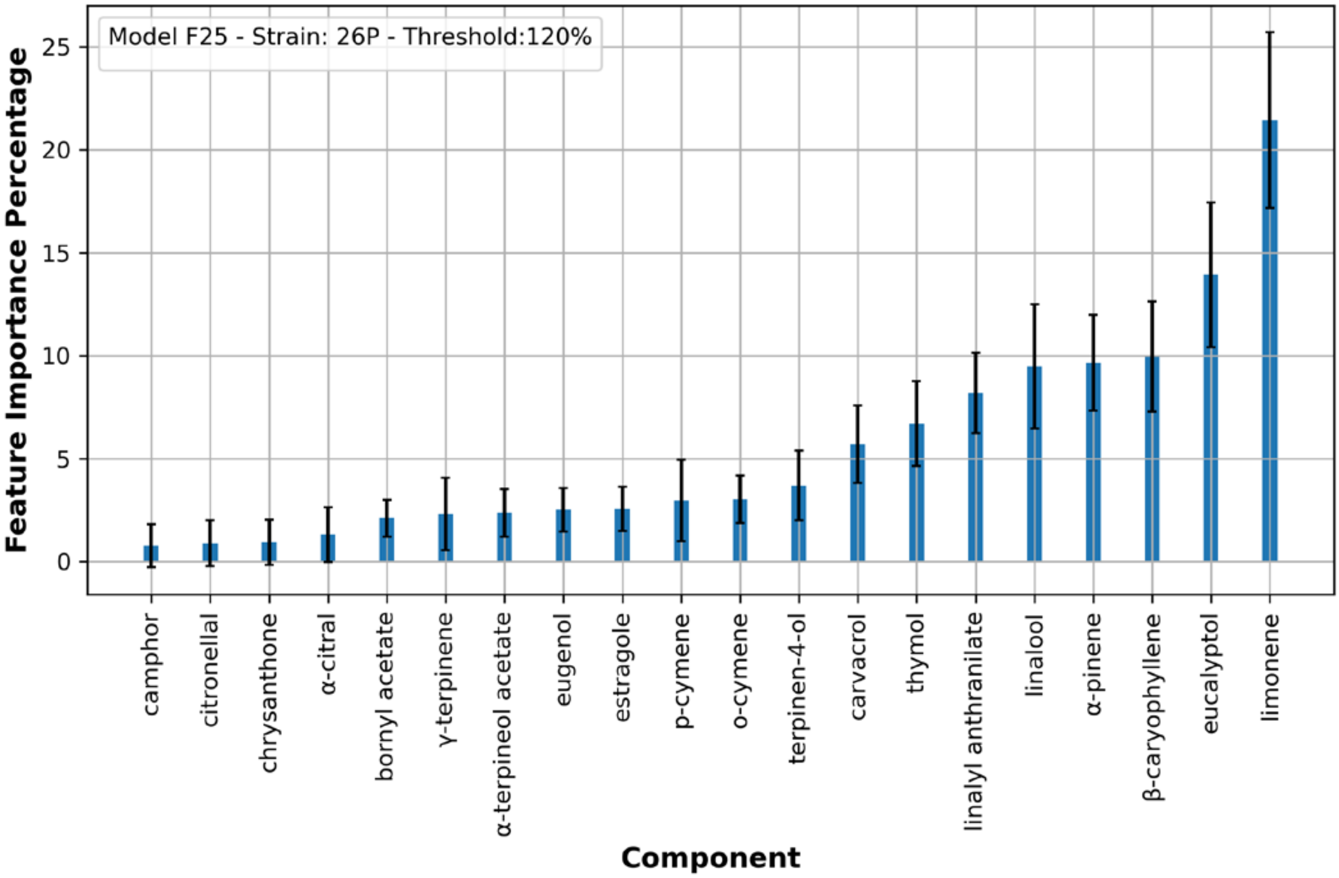

Figure S10. Feature importance for model F10 (see main text Table 1). The top 20 components are displayed.

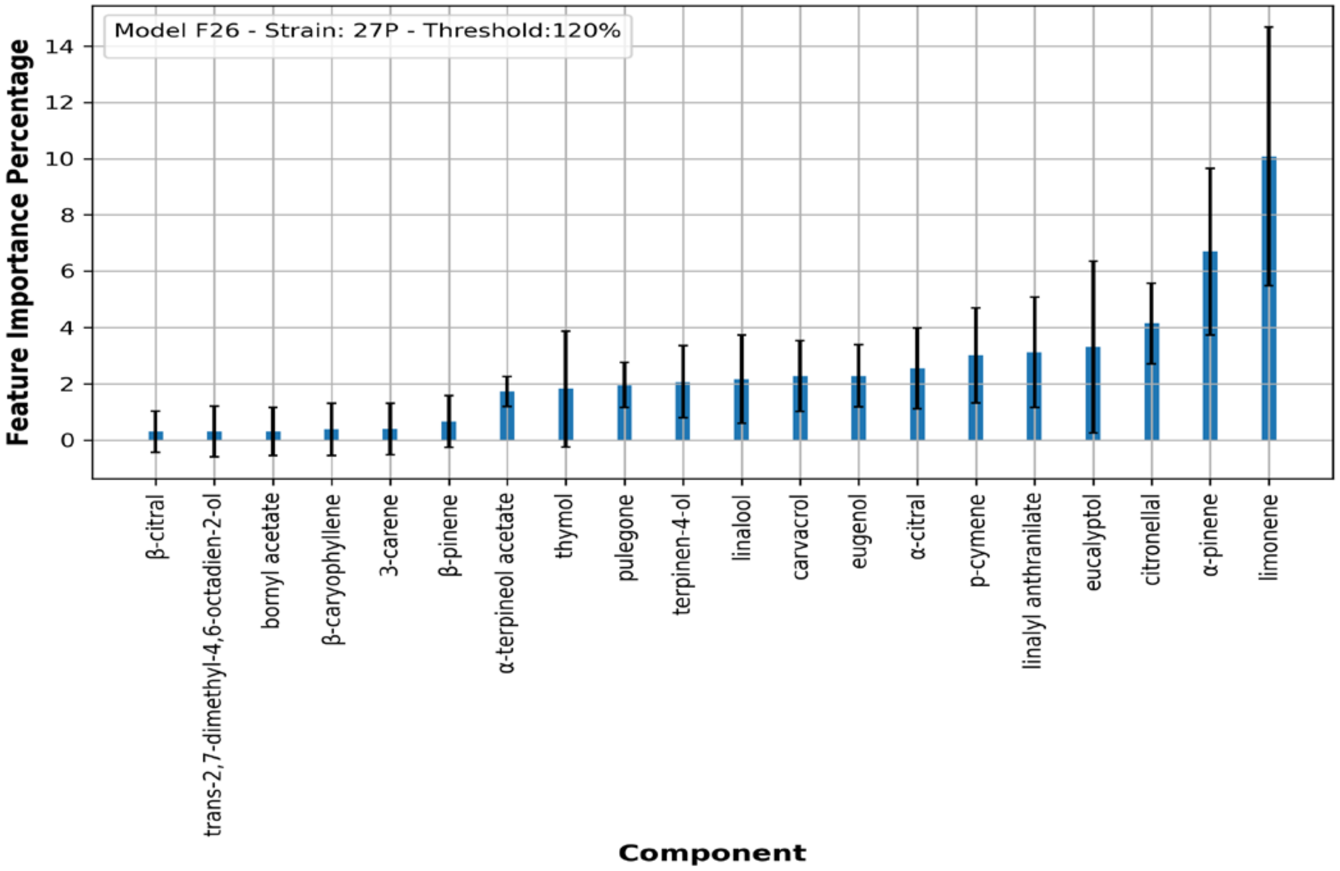

Figure S11. Feature importance for model F11 (see main text Table 1). The top 20 components are displayed.

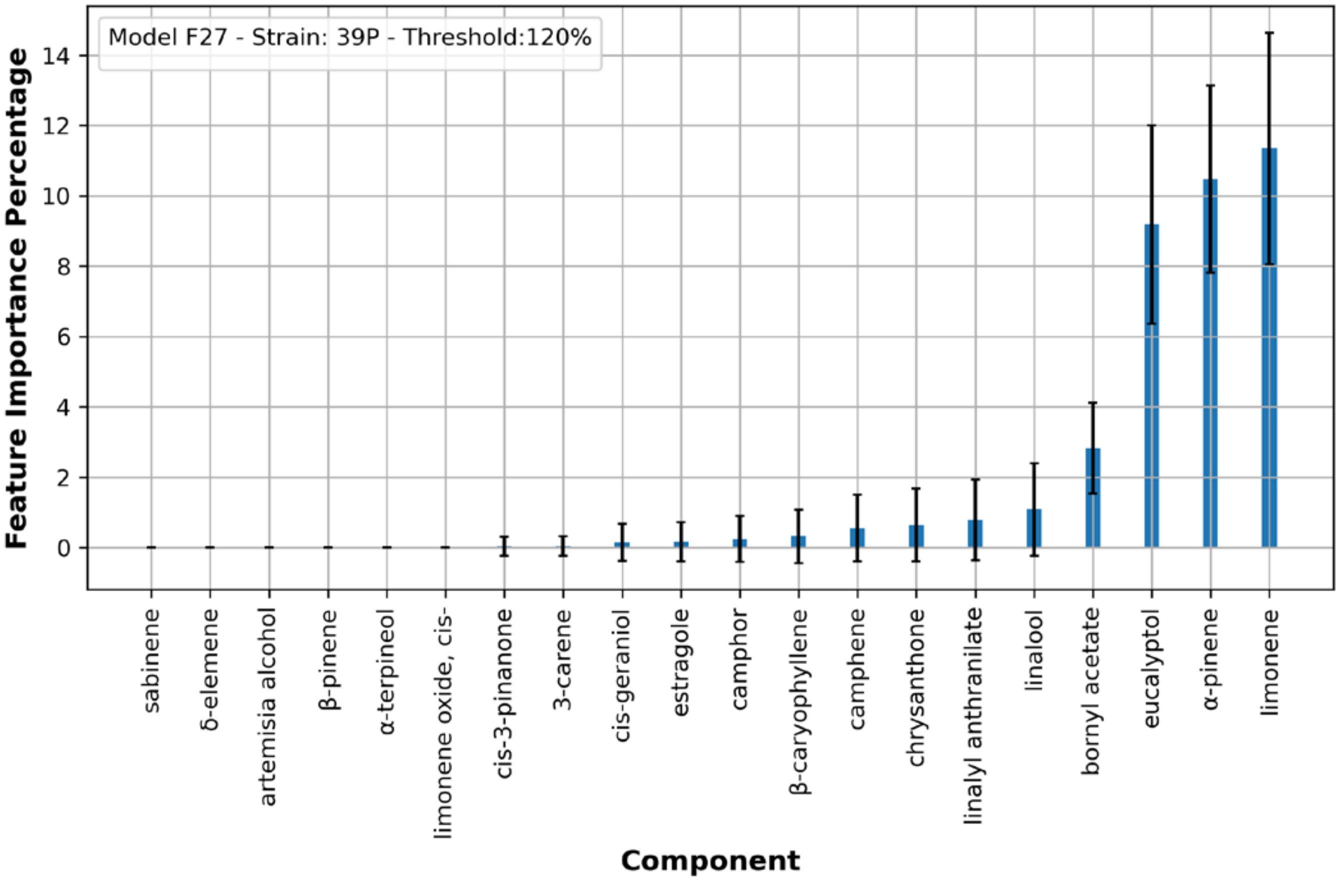

Figure S12. Normalized feature importances for the final models F1-F6 developed at threshold value of 40% (see main text Table 1). The top 20 components are displayed.

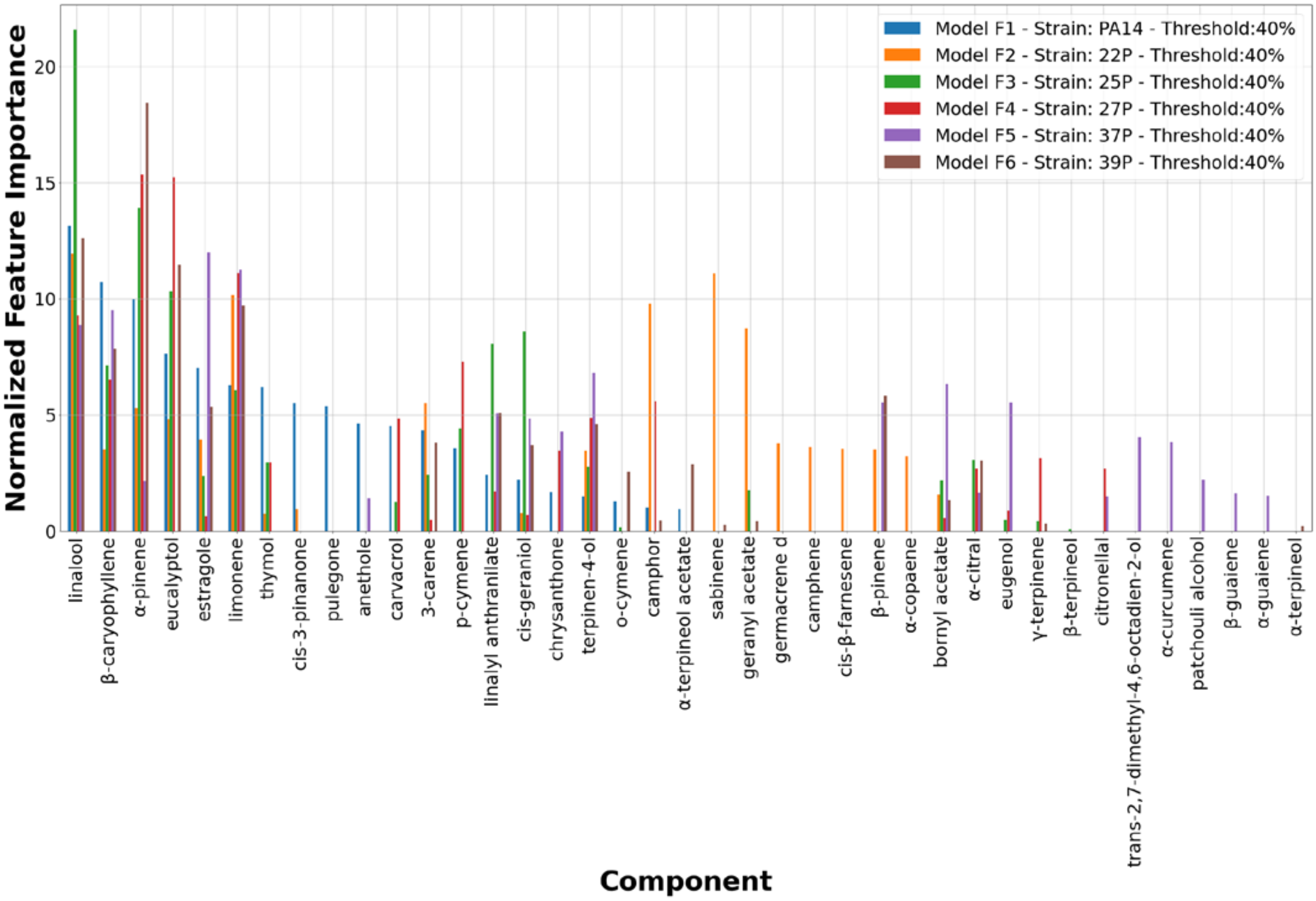

Figure S13. Normalized feature importances for the final models F7-F11 developed at threshold value of 120% (see main text Table 1). The top 20 components are displayed.

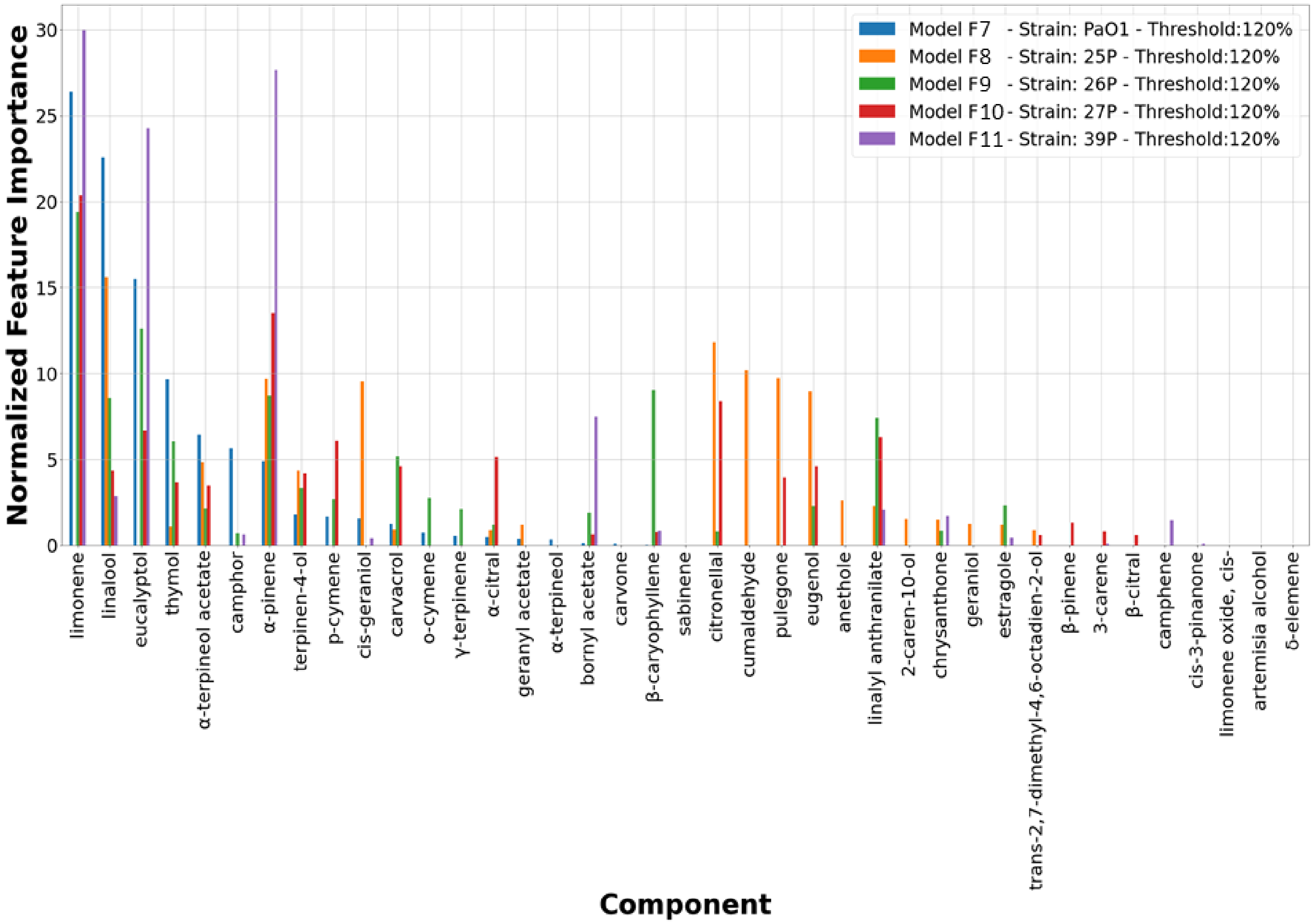

Supplement: Supplementary file 1 [file microorganisms-10-00887-s001.zip › microorganisms-1684730-supplementary.pdf]
